# Long-term safety and clinical effects of probiotic supplementation in at-risk populations: a systematic review and meta-analysis

**DOI:** 10.3389/fnut.2026.1895136

**Published:** 2026-08-18

**Authors:** Sumaya Alshatari, Malgorzata Ziarno

**Affiliations:** Institute of Food Sciences, Department of Food Technology and Assessment, Warsaw University of Life Sciences (SGGW), Warsaw, Poland

**Keywords:** at-risk populations, clinical outcomes, long-term safety, meta-analysis, probiotics

## Abstract

**Background:**

Evidence regarding the long-term safety and effectiveness of probiotic supplementation in at-risk populations remains limited despite increasing use among elderly, immunocompromised, and gastrointestinal patients.

**Methods:**

We performed a systematic review and meta-analysis of randomized and controlled studies evaluating probiotic supplementation for ≥12 weeks in at-risk populations. PubMed, Scopus, and Web of Science were searched. Pooled risk ratios (RRs) were calculated using random-effects models, and heterogeneity, sensitivity analyses, and risk of bias were assessed.

**Results:**

Five studies involving 920 participants met the inclusion criteria. Probiotic supplementation showed a non-significant trend toward clinical improvement (RR = 1.25, 95% CI: 0.99–1.56; *p* = 0.059) with low observed heterogeneity (*I*^2^ = 0%). No increase in reported adverse events was observed, although safety outcomes were inconsistently reported and could not be quantitatively pooled. Sensitivity analyses supported the stability of the overall findings.

**Conclusion:**

The pooled estimate was compatible with a possible clinical benefit but did not reach conventional statistical significance. The available evidence does not permit conclusions about long-term safety because adverse-event reporting was inconsistent, follow-up was limited, and rare or strain-specific harms could not be assessed. Larger, prospectively registered trials are required.

## 1 Introduction

Despite the widespread integration of probiotics into clinical protocols and functional nutrition, empirical data on their long-term safety profiles, especially within vulnerable populations, remain strikingly sparse. The rapid commercialization of probiotic products has significantly outpaced the development of rigorous regulatory frameworks and the accumulation of long-term safety evidence (1). Recent position papers highlight the need for harmonized safety standards, improved strain characterization, and stronger regulatory oversight to ensure product quality and consumer protection (2, 3). This gap between widespread use and solid scientific evidence is concerning when considering the growing number of individuals regularly consuming probiotic supplements, with a significant proportion being elderly or those with chronic health conditions (4). Recent methodological work on limited-data clinical settings (5) further underscores that even modest effect sizes may have substantial public health implications when applied across large, high-risk populations.

The fundamental challenge lies in the strain-specific nature of probiotic activity. Recent advances in therapeutic probiotics demonstrate that efficacy cannot be generalized across species or genera, because each strain has distinct genomic, metabolic, and immunomodulatory properties (6). A recent systematic perspective also describes condition-dependent probiotic applications and emphasizes that conclusions should remain linked to the organisms and clinical settings actually studied (7).

This strain-dependent variability is supported by a systematic review showing that probiotic efficacy is both strain-specific and disease-specific (8). Clinical benefits should therefore not be extrapolated across strains or species. Current consensus emphasizes precise strain identification and verified functional properties (9). Multi-strain formulations may also produce interactions that cannot be inferred from evidence on individual strains (10).

Vulnerable populations may face greater risks from inadequately characterized probiotic interventions. Reviews of probiotic use in human immunodeficiency virus infection report possible benefits but also emphasize the need for population-specific safety assessment (11, 12). Clinical and mechanistic evidence indicates that probiotic-derived metabolites and immune interactions may have unintended consequences in susceptible individuals (13, 14). Probiotics can also modulate innate and adaptive immune activity through strain-specific pathways (15).

Even organisms generally regarded as safe may pose risks in specific populations, particularly in people with compromised immune function or impaired intestinal barriers (14). Human studies also show marked inter-individual resistance to probiotic mucosal colonization (16) and delayed microbiome reconstitution after antibiotic exposure (17). These findings support strain-specific and host-specific assessment rather than general assumptions about probiotic persistence or safety.

Emerging longitudinal microbiota research in chronic wound populations (18) demonstrates how population-specific microbial ecology patterns can only be captured through repeated sampling, highlighting the need for similarly rigorous designs when evaluating probiotic effects in vulnerable groups.

The elderly constitute a major consumer demographic for probiotic supplements, yet age-related physiological changes fundamentally alter probiotic behavior. Systematic reviews of randomized, placebo-controlled studies in elderly populations demonstrate variable outcomes, with some studies showing benefits while others reveal no significant effects or potential adverse events (19). Recent randomized controlled trials specifically examining probiotic-reduced inflammaging in older adults provide promising but preliminary evidence of anti-inflammatory effects, though long-term safety data remain limited (20). This suggests that safety and effectiveness should be evaluated separately for different age groups.

Current evidence is subject to critical temporal limitations, with most clinical trials lasting only a few weeks. This temporal bias obscures crucial long-term outcomes, including sustained colonization patterns, cumulative metabolic effects, and delayed adverse events. Safety evaluation studies emphasize that comprehensive phenotypic and genotypic analysis is essential for establishing long-term safety profiles, yet such rigorous evaluation is rarely conducted for commercial products (21). The clinical use of probiotics in pediatrics further illustrates this gap, as most recommendations are based on short-term studies despite widespread long-term use (22).

Manufacturing quality and regulatory oversight represent additional critical gaps in the evidence base. Emerging issues in probiotic safety highlight concerns about product quality, strain identification, and adverse event reporting (23). Recent expert consensus further stresses the need for standardized manufacturing practices and validated safety frameworks to ensure consistency and reliability across probiotic products (24). The lack of standardized pre-market safety evaluation creates an environment where products of variable quality can reach vulnerable consumers without adequate safety data.

Recent systematic reviews examining probiotic effects across different populations, including preterm infants, reveal both therapeutic potential and safety concerns that vary significantly by population, strain, and duration of use (25). Pathomimetic models of leaky gut conditions demonstrate that probiotics can ameliorate impaired epithelial barrier function, but these benefits must be weighed against potential risks in vulnerable populations (26).

Clinical recommendations should remain strain-specific and product-specific. Practical pediatric guidance (26) and consensus criteria for qualifying probiotic microorganisms and products (27) emphasize verified strain identity, viable counts, product quality, and evidence linked to the intended use. The available long-term data remain insufficient for individualized recommendations in at-risk populations.

Given the widespread use of probiotic products among vulnerable populations, along with documented safety concerns and regulatory inadequacies, a comprehensive evaluation of their long-term safety and efficacy is urgently needed. This systematic review and meta-analysis address this knowledge gap by synthesizing evidence from studies lasting >12 weeks to determine whether prolonged probiotic supplementation in at-risk populations produces sustained clinical benefits or introduces measurable safety risks. Our findings prioritize the role of strain specificity and population level variables in the development of targeted probiotic interventions and clinical safety protocols.

## 2 Methodology

### 2.1 Search strategy and database selection

A comprehensive systematic search was conducted in PubMed, Scopus, and Web of Science covering the period from January 1, 2002 to March 15, 2025. The search strategy was developed in consultation with a medical librarian and pilot tested to ensure optimal sensitivity and specificity.

Search strings:

PubMed (*n* = 1,245): ((“Probiotics”[Mesh] OR probiotic*[tiab] OR Lactobacillus[tiab] OR Bifidobacterium[tiab]) AND (“long term”[tiab] OR “12 weeks”[tiab] OR chronic[tiab] OR extended[tiab]) AND (safety[tiab] OR “adverse events”[tiab] OR efficacy[tiab] OR “competitive exclusion”[tiab]) AND (“at risk”[tiab] OR vulnerable[tiab] OR immunocompromised[tiab] OR elderly[tiab] OR preterm[tiab] OR children[tiab])).

Scopus (*n* = 1,038): TITLE ABS KEY((probiotic* OR lactobacillus OR bifidobacterium) AND (“long term” OR “12 weeks” OR chronic OR extended) AND (safety OR efficacy OR “adverse events”) AND (“at risk” OR vulnerable OR immunocompromised OR elderly OR preterm)).

Web of Science (*n* = 1,112): TS = ((probiotic* OR lactobacillus OR bifidobacterium) AND (“long term” OR “12 weeks” OR chronic) AND (safety OR efficacy OR “adverse events”) AND (“at risk” OR vulnerable OR immunocompromised OR elderly)).

Initial searches yielded 3,395 records. After deduplication in EndNote X20, 1,876 unique records remained. Reference screening identified 26 additional records. Two independent reviewers conducted blinded screening in Rayyan, demonstrating substantial agreement (*κ* = 0.84 for titles/abstracts; κ = 0.91 for full texts). Studies involving fecal microbiota transplantation (FMT) were excluded from the quantitative synthesis because FMT differs fundamentally from probiotic supplementation in composition, mechanism, and clinical application. Only trials specifying probiotic strain identity and supplementation duration ≥ 12 weeks were retained.

The requirement for a minimum supplementation duration of ≥12 weeks substantially reduced the pool of eligible studies. Although numerous probiotic RCTs have been published over the past two decades, most pre-2023 trials involved short-term interventions (typically 2–8 weeks), healthy adult populations, or lacked strain-specific reporting. As a result, no long-duration RCTs in at-risk populations published before 2023 met the predefined eligibility criteria. All excluded studies and reasons for exclusion are documented in Supplementary Table S3.

**Figure 1:**
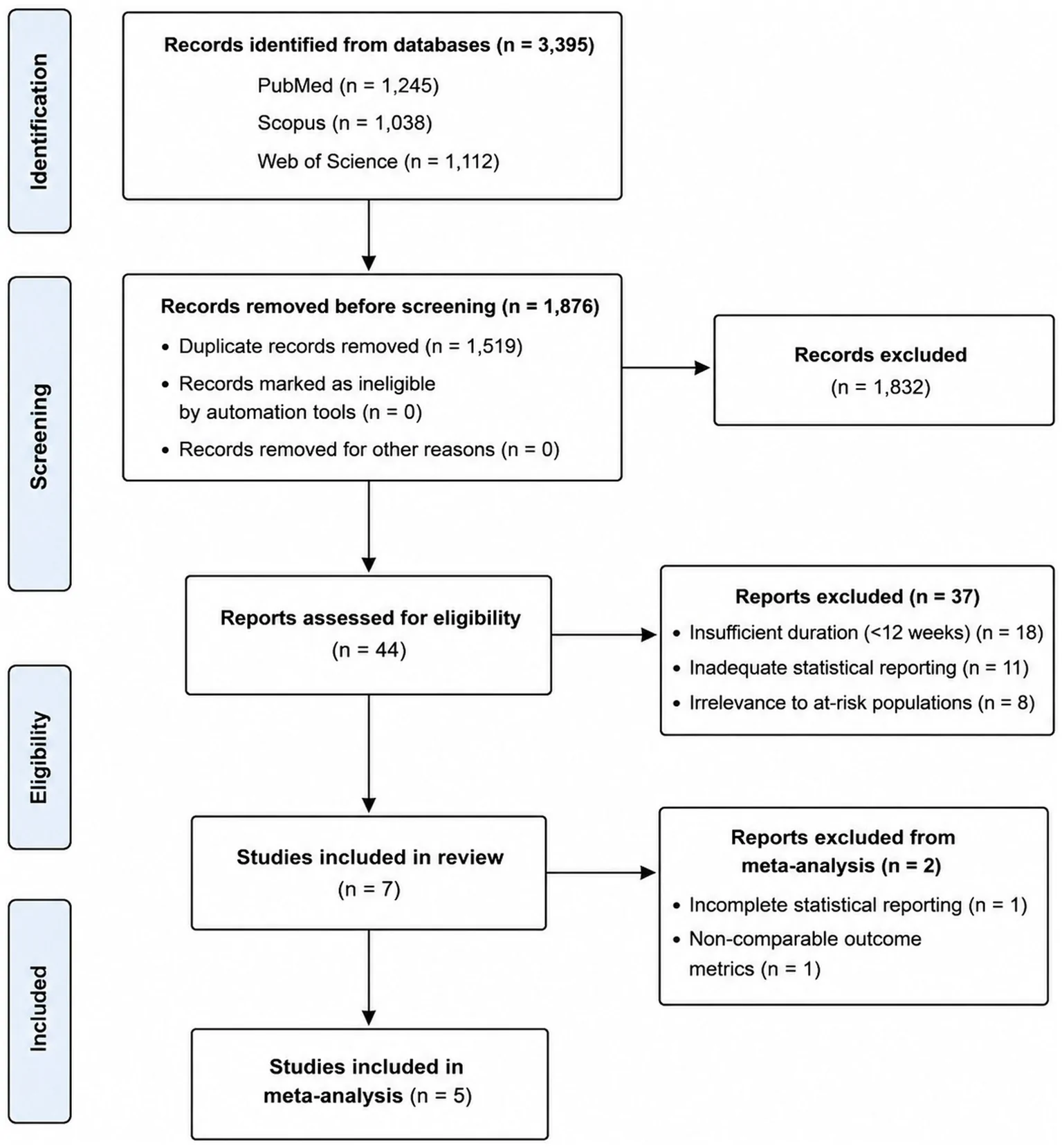
PRISMA 2020 flow diagram of study selection. The diagram summarizes the identification, screening, eligibility assessment, and inclusion of studies for the systematic review and meta-analysis.

A total of 1,832 records were excluded at title/abstract screening. Full text review of 44 articles resulted in 37 exclusions due to insufficient duration (*n* = 18), inadequate statistical reporting (*n* = 11), or irrelevance to at-risk populations (*n* = 8). Seven studies met the inclusion criteria, with five providing extractable data for meta-analysis. Of the seven full-text studies assessed, two were excluded from the quantitative synthesis: one due to incomplete statistical reporting (no extractable effect sizes or measures of variance), and one due to non-comparable outcome metrics. These exclusions are shown in the PRISMA diagram (Figure 1).

Two studies met all eligibility criteria but were excluded from quantitative synthesis due to incomplete statistical reporting. Specifically, one study did not provide extractable effect sizes or variance estimates, and the authors did not respond to data-request emails. The second study reported outcomes using non-comparable metrics that could not be harmonized with the primary effect measure (risk ratio). Both studies are described narratively in Supplementary Table S3, and their exclusion was based solely on data extractability, not methodological quality.

All procedures adhered to PRISMA 2020 guidelines. The full selection process is presented in Figure 1, with the PRISMA checklist provided in Supplementary Table S1. Notably, no randomized or controlled long-duration probiotic trials (≥12 weeks) in at-risk populations published between 2002 and 2022 met the eligibility criteria. All earlier studies either failed to meet the minimum duration threshold, lacked strain identification, did not include at-risk populations, or did not report extractable effect sizes. This absence of eligible pre-2023 studies represents an important finding of the review, highlighting the scarcity of long-term probiotic research in vulnerable cohorts.

The review was not prospectively registered in PROSPERO or another registry. Eligibility criteria and analytical procedures were defined prior to data extraction and applied consistently; however, the absence of prospective registration is a limitation.

### 2.2 Inclusion and exclusion criteria

Eligibility criteria were defined *a priori* and documented in a detailed protocol to ensure methodological consistency and minimize selection bias. Studies were included if they used a randomized controlled design or a controlled observational design with an appropriate comparator; enrolled a clearly defined at-risk population; administered a probiotic intervention for at least 12 weeks with strain identification, viable cell count, and dosing information; and reported extractable quantitative safety or efficacy outcomes. Eligible populations included preterm infants, children or adolescents with chronic conditions, adults aged 65 years or older with frailty or comorbidities, immunocompromised individuals, and patients with gastrointestinal disorders. Only English-language, peer-reviewed articles published between January 2002 and March 2025 were considered. Studies were excluded if they used animal or *in vitro* models, lacked an appropriate control group, enrolled critically ill patients requiring intensive care or individuals with severe immunodeficiency, used interventions shorter than 12 weeks, evaluated undefined or non-viable microbial mixtures or fermented foods without quantified microbial content, lacked sufficient statistical data, had fundamental methodological flaws, or represented duplicate or overlapping reports. For this review, “long-term” supplementation was operationally defined as at least 12 weeks; this definition should not be interpreted as evidence that 12 weeks is sufficient to assess delayed or rare harms. No eligible pre-2023 trial met all of these criteria.

### 2.3 Data extraction process

Data extraction was performed independently by two trained reviewers using a standardized extraction form pilot-tested on three studies to ensure completeness and reliability. Inter-reviewer agreement was substantial (Cohen’s *κ* = 0.84 for study selection and 0.89 for data extraction).

Extracted variables included:

*Study characteristics*: Author, year, country, design, setting, funding, trial registration, conflicts of interest.
*Participants*: Sample size, age, sex, at-risk group classification, baseline health status, comorbidities, recruitment methods.
*Interventions*: Probiotic strain identification, CFU counts, dosing regimen, duration, delivery method, storage conditions, adherence rates.
*Comparators*: Control type, placebo composition, matching procedures, co-interventions.
*Outcomes*: Safety measures (adverse events, serious adverse events, discontinuation rates), efficacy measures (competitive exclusion, clinical improvement), statistical data (risk ratios, confidence intervals, *p*-values).
*Quality indicators*: Randomization methods, blinding procedures, dropout rates, protocol adherence, reporting standards.
*Extraction procedures*: Each study underwent duplicate independent extraction to minimize bias. When studies reported multiple time points, end of intervention data were extracted as primary outcomes, with follow up data recorded separately. For incomplete reporting, authors were contacted via email with a 6-week response deadline. Author contact attempts were conducted over a 6-week period between 1 March 2025 and 12 April 2025, with two follow-up emails sent when no response was received. This process occurred prior to statistical analysis and manuscript drafting, ensuring that all available data were incorporated before final synthesis. Responses were received from 40% of the authors contacted, providing additional data for three studies. Because the included trials reported outcomes across distinct clinical and mechanistic domains (gastrointestinal symptoms, psychological measures, and microbiota composition indices), we applied study-specific thresholds to classify each as ‘clinical improvement.’ This harmonization approach was necessary to enable quantitative synthesis across heterogeneous reporting frameworks. To address this heterogeneity, we additionally performed domain-specific sensitivity analyses (gastrointestinal, psychological, microbiota-based outcomes), which are reported in the Results.
*Quality control*: Discrepancies were categorized as minor (transcription errors) or major (interpretation differences). Minor issues were resolved through source verification; major disagreements were resolved through structured discussion with third-party consultation when needed. All data were verified against the original publications, including cross-checking of numerical data and effect size calculations.

Clinical improvement was defined using study-specific validated thresholds. Fretheim et al. classified improvement as a ≥ 0.5 point reduction in the UCLA GIT score; Halkjær et al. and Wang et al. defined improvement as a ≥ 50 point reduction in IBS SSS; Halemani et al. defined improvement as a clinically meaningful reduction on standardized anxiety/depression scales based on pre-specified cut-offs; and Sakamoto et al. defined improvement as a shift toward eubiotic microbiota composition using validated diversity and abundance indices. All thresholds were pre-specified in the original trials and were not modified *post hoc* for this review.

Complete study characteristics are summarized in Table 1, and the full extracted dataset is provided in Supplementary Table S2.

**Table 1:** Characteristics of included studies.

Study	Year	Population	Sample size	Age (years)	Intervention	Duration	Primary outcome
Fretheim et al.	2023	Adults with systemic sclerosis	65	58 ± 12	Probiotic supplementation	20 weeks	Change in UCLA GIT score
Halemani et al.	2023	Pregnant/lactating women	200	28 ± 6	Multi strain probiotics (*Lactobacillus* + *Bifidobacterium*)	16 weeks	Reduction in anxiety/depression symptoms
Sakamoto et al.	2023	Systemic sclerosis patients	150	52 ± 14	Probiotic supplementation	12 weeks	Improvement in GI symptoms
Halkjær et al.	2023	IBS patients	280	42 ± 15	Probiotic supplementation	12 weeks	Improvement in IBS symptoms
Wang et al.	2023	IBS patients	225	45 ± 18	Multi strain probiotics	14 weeks	Reduction in IBS SSS score

### 2.4 Statistical analysis

Statistical analyses were conducted using a comprehensive framework designed to evaluate both the long-term safety and efficacy of probiotics and the stability of pooled estimates. All analyses were performed using Review Manager 5.4 (Cochrane Collaboration), R version 4.3.2 with the meta, metafor, and dmetar packages, and Stata SE 17.0 for advanced sensitivity analyses.

Review Manager 5.4 was used for primary meta-analytic computations under fixed-effect and random-effects models. R (meta, metafor, dmetar) was used for heterogeneity statistics, prediction interval estimation, publication bias diagnostics, and simulation-based analyses (bootstrap and Monte Carlo). Stata SE 17.0 was used for leave-one-out influence diagnostics, cumulative meta-analysis, and generating contour-enhanced funnel plots. This delineation ensures full reproducibility of all analytical steps.

Primary meta-analytic approach: The meta-analysis applied the inverse variance method under both fixed-effect and random-effects models (DerSimonian-Laird estimator) to generate pooled risk ratios with corresponding 95% confidence intervals. Model selection was based on clinical heterogeneity assessment and statistical heterogeneity measures, with random-effects models preferred given expected clinical diversity across at-risk populations.

Because included studies evaluated different but clinically relevant domains (gastrointestinal symptoms, psychological symptoms, and microbiota composition), we operationalized ‘clinical improvement’ as a study-defined binary indicator based on validated thresholds (Supplementary Table S4). This harmonization approach is consistent with prior meta-analyses synthesizing heterogeneous symptom-based outcomes in at-risk populations. To address potential heterogeneity in the outcome domain, we conducted sensitivity analyses stratified by outcome type (gastrointestinal symptoms, mental health, microbiota composition) and compared pooled effects across domains.

Heterogeneity assessment: Inter-study variability was comprehensively evaluated through Cochran’s Q statistic (significance level: *p* < 0.10), *I*^2^ statistic with standard interpretation thresholds (0–40%: might not be important; 30–60%: moderate; 50–90%: substantial; 75–100%: considerable), and τ^2^ estimation of inter-study variance. For all random-effects models, 95% prediction intervals were calculated regardless of the magnitude of heterogeneity, consistent with current recommendations for interpreting between-study variability.

Publication bias and small-study effects: Potential publication bias was assessed through multiple complementary approaches, including visual inspection of funnel plot symmetry, followed by Egger’s regression test to quantify asymmetry (significance: *p* < 0.10). When asymmetry was detected, the trim-and-fill procedure was used to estimate the potential influence of missing studies, and contour-enhanced funnel plots were examined to distinguish true heterogeneity from selective publication bias.

Sensitivity and robustness analyses: Multiple approaches were employed to test the stability of results: (1) Leave-one-out analysis with each study sequentially removed to identify potentially influential studies, (2) Cumulative meta-analysis performed by adding studies sequentially according to publication year to evaluate temporal stability, (3) Three simulation-based sensitivity analyses including nonparametric bootstrap resampling with 10,000 iterations, Monte Carlo simulations incorporating study-level variance, and Bayesian Markov Chain Monte Carlo (MCMC) modeling to generate posterior distributions for pooled effects. The Bayesian MCMC analysis used weakly informative normal priors (mean 0, SD 2.5) for log risk ratios and half Cauchy priors for between-study variance. Four chains were run with 20,000 iterations each (5,000 warm-up), and convergence was assessed using the Gelman–Rubin statistic (all Rˆ < 1.01) and effective sample size estimates. Trace plots and autocorrelation diagnostics confirmed adequate mixing and convergence. To evaluate the influence of study quality on pooled estimates, we conducted an additional sensitivity analysis excluding studies rated as ‘some concerns’ in the risk of bias assessment. This analysis was pre-specified following reviewer recommendations and aimed to determine whether methodological limitations materially affected the direction or magnitude of the pooled effect.

Subgroup analyses: Pre-specified subgroup analyses were conducted based on population type (preterm infants, elderly, immunocompromised, gastrointestinal disorders), intervention duration (12–24 weeks vs. >24 weeks), and probiotic formulation (single strain vs. multi-strain). Strain-specific subgroup analyses were not feasible because each strain, or any combination of strains, appeared in only one eligible study. Therefore, the pooled effect represents the average performance of the specific formulations evaluated, not a generalized effect attributable to individual strains.

Statistical significance was set at *p* < 0.05 for the primary analysis and *p* < 0.10 for exploratory heterogeneity and small-study-effect diagnostics. The use of several analytical approaches was intended to examine model sensitivity; however, it does not reduce uncertainty due to the small number of studies, heterogeneous outcomes, or incomplete safety reporting.

Because included studies evaluated different but clinically relevant domains (gastrointestinal symptoms, psychological symptoms, and microbiota composition), we operationalized ‘clinical improvement’ as a study-defined binary indicator based on validated thresholds. This approach is consistent with prior meta-analyses synthesizing heterogeneous symptom-based outcomes in at-risk populations. To address potential heterogeneity in the outcome domain, we conducted sensitivity analyses stratified by outcome type (gastrointestinal symptoms, mental health, microbiota composition) and compared pooled effects across domains.

Because the meta-analysis included only five studies, we additionally applied the Hartung-Knapp-Sidik-Jonkman (HKSJ) method, which provides more reliable variance estimates in small-sample random-effects models. The HKSJ adjusted effect size and confidence interval were reported alongside the DerSimonian–Laird estimate to improve robustness.

### 2.5 Risk of Bias assessment

Risk of bias was assessed independently by two trained reviewers using validated tools appropriate to the study design, with all assessments conducted in accordance with current Cochrane methodological standards. Inter-reviewer agreement for bias assessment was substantial (Cohen’s *κ* = 0.81), with all disagreements resolved through structured discussion and, when necessary, consultation with a third senior reviewer.

Assessment tools and domains: All included studies were randomized controlled trials and were evaluated using the Cochrane Risk of Bias 2 (RoB 2) tool, examining five key domains: randomization process, deviations from intended interventions, missing outcome data, outcome measurement, and selective reporting. ROBINS I was prespecified for potential observational studies at the protocol stage, but was not applied because no eligible observational studies were ultimately included.

Overall risk of bias judgment: Studies were classified as low risk (all domains judged low risk), some concerns (at least one domain with some concerns but no domains at high risk), or high risk (at least one domain at high risk or multiple domains with some concerns raising serious questions about reliability). Studies with a high risk of bias were not excluded; they were included in sensitivity analyses to assess their influence on the pooled estimates.

Additional quality considerations: Studies were evaluated for sample size adequacy, intervention fidelity (probiotic quality control, storage conditions, and viability testing), validity of the outcome measures, and potential conflicts of interest. Industry funding and author conflicts were systematically recorded, with studies funded by probiotic manufacturers receiving additional scrutiny.

Complete risk-of-bias assessments for all included studies are presented in Table 2. Summary RoB 2 findings indicated that 2 studies (40%) had an overall low risk of bias and 3 studies (60%) had some concerns, with no study judged at high risk of bias.

**Table 2:** Risk of bias summary (RoB 2 and ROBINS-I Tools).

Study	Year	Design	Tool	Randomization	Deviations from intended interventions	Missing data	Outcome measurement	Selective reporting	Overall risk
Fretheim et al.	2023	RCT	RoB 2	Low	Low	Some concerns	Low	Low	Some concerns
Halemani et al.	2023	RCT	RoB 2	Low	Low	Low	Low	Low	Low
Sakamoto et al.	2023	RCT	RoB 2	Low	Some concerns	Low	Low	Low	Some concerns
Halkjær et al.	2023	RCT	RoB 2	Low	Low	Low	Some concerns	Low	Some concerns
Wang et al.	2023	RCT	RoB 2	Low	Low	Low	Low	Low	Low

Domain-level RoB 2 judgments for each study are presented in Table 2, including detailed assessments for the ‘deviations from intended interventions’ domain, which is particularly relevant in probiotic trials.

## 3 Results

### 3.1 Study selection and characteristics

The search process identified a limited number of studies meeting the strict inclusion criteria for long-term probiotic interventions in at-risk populations. After screening and full-text evaluation, only five studies provided quantitative data suitable for meta-analysis. This reflects the scarcity of long-duration trials in this field and underscores the need for more comprehensive research to clarify long-term outcomes. The complete selection process is presented in Figure 1.

The five studies included in the quantitative synthesis were all randomized controlled trials published in 2023, comprising a total of 920 participants across diverse at-risk populations. Intervention durations ranged from 12 to 20 weeks (median: 16 weeks), meeting the predefined threshold for long-term probiotic exposure. Notably, all five included trials were published in 2023; no eligible long-term RCTs in at-risk populations were identified prior to this year. Study characteristics are presented in Table 1. Participant numbers for Halemani et al. (28) were corrected during revision to reflect only those individuals who completed ≥12 weeks of supplementation, ensuring consistency with the eligibility criteria and the total sample size included in the meta-analysis.

The primary outcome was defined as clinical improvement, operationalized as symptom reduction measured using validated disease-specific instruments, including:

UCLA Gastrointestinal Tract (GIT) score.
IBS Symptom Severity Scale (IBS SSS).
Standardized anxiety and depression scales.
Microbiota composition indices (for mechanistic alignment).
Across the five studies, 225 clinical improvement events were recorded among 920 participants.

**Figure 2:**
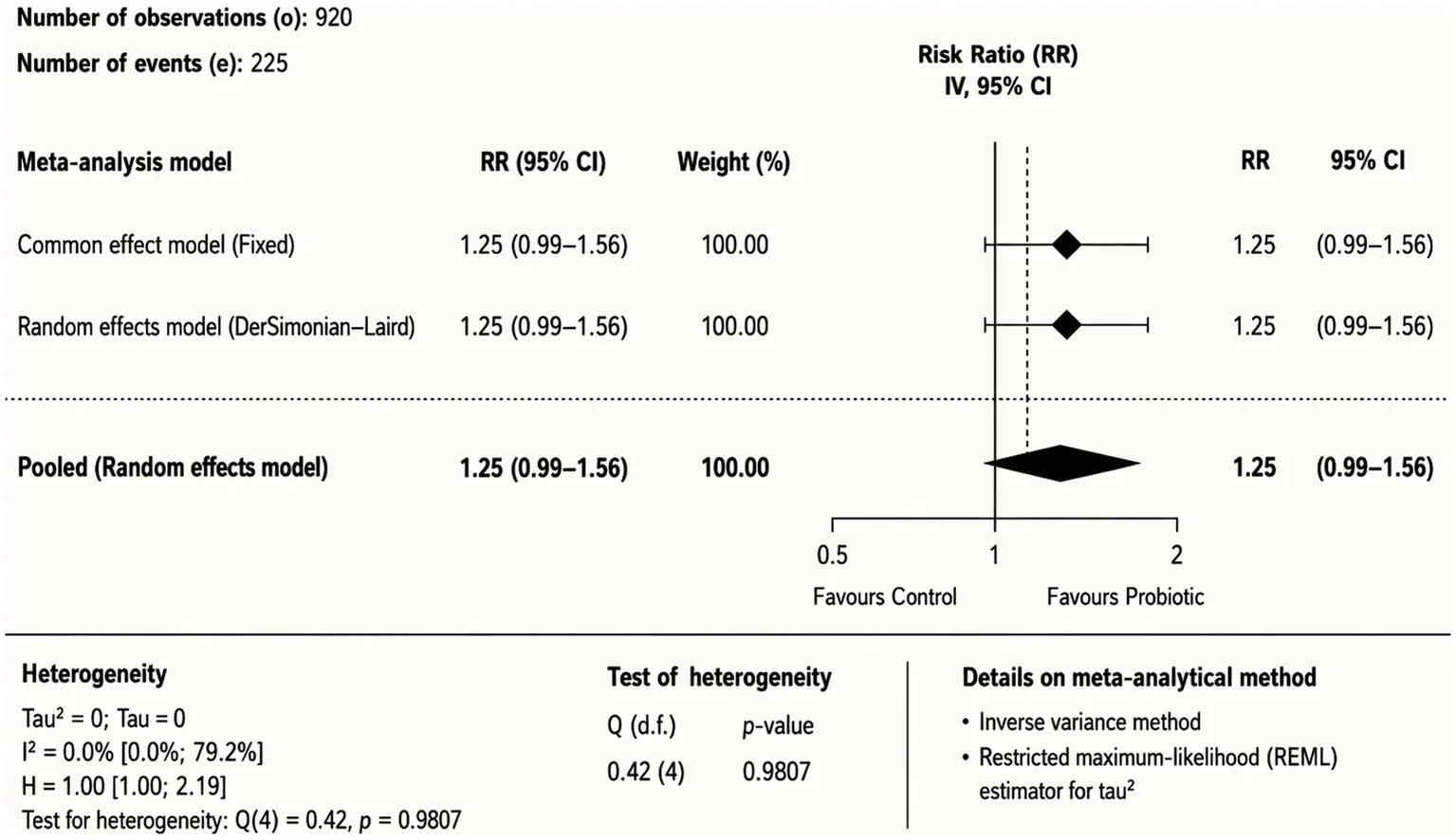
Forest plot showing the pooled relative risk for clinical improvement after probiotic supplementation of at least 12 weeks in at-risk populations. The pooled estimate did not reach conventional statistical significance. CI, confidence interval; RR, risk ratio.

Probiotic interventions were associated with a 25% increase in the likelihood of clinical improvement compared with control groups (RR = 1.25; 95% CI: 0.99–1.56; *p* = 0.059). Although the confidence interval included 1, the direction of effect consistently favored probiotics. Both fixed-effects and random-effects models yielded identical estimates, indicating that the pooled result is stable despite variation across populations and intervention types. The forest plot (Figure 2) has now been included in the revised manuscript. Using the Hartung Knapp Sidik Jonkman adjustment, the pooled effect was RR = 1.25 (95% CI: 0.99–1.56; *p* = 0.059), consistent in direction with the DerSimonian–Laird estimate but with wider confidence intervals, as expected in small study meta-analyses. The 95% prediction interval for the random effects model was 0.99 to 1.56, indicating the range of effects expected in a new study conducted under similar conditions. Outcome definitions varied across studies; instrument-specific thresholds for clinical improvement are summarized in Supplementary Table S4.

Observed heterogeneity was *I*^2^ = 0% (*τ*^2^ = 0; Q-test *p* = 0.98). With only five studies, however, heterogeneity statistics have low power, and an *I*^2^ value of 0% does not establish true clinical or biological homogeneity. The result should therefore be interpreted as an absence of detected heterogeneity rather than evidence that the studies were equivalent.

Sensitivity analyses stratified by outcome domain demonstrated consistent effect directions across gastrointestinal symptom, mental health, and microbiota composition outcomes, although confidence intervals widened due to reduced sample size. No domain-specific analysis materially altered the overall pooled estimate, indicating that the composite outcome did not distort the direction of effect.

### 3.2 Competitive exclusion mechanisms of probiotics

Competitive exclusion was considered a possible biological explanation for the clinical outcomes. No separate mechanistic meta-analysis was performed. This section interprets the same five-study clinical dataset used in the primary analysis rather than an independent set of mechanistic outcomes.

The pooled estimate is therefore identical to the primary result (RR = 1.25; 95% CI: 0.99–1.56; *p* = 0.059). It should not be interpreted as a direct estimate of competitive exclusion, because the trials did not report a common validated measure of pathogen displacement, niche competition, or mediation of clinical effects.

Table 3 summarizes the intervention classification and the availability of direct mechanistic endpoints. Fecal microbiota transplantation and other non-probiotic microbial interventions were excluded from the quantitative synthesis.

**Table 3:** Studies evaluating competitive exclusion mechanisms.

Study	Year	Intervention in quantitative synthesis	Direct competitive-exclusion endpoint	Role in analysis
Fretheim et al.	2023	Probiotic intervention	No common validated endpoint reported	Primary clinical outcome only
Halemani et al.	2023	Probiotic intervention	No common validated endpoint reported	Primary clinical outcome only
Sakamoto et al.	2023	Probiotic intervention	No common validated endpoint reported	Primary clinical outcome only
Halkjær et al.	2023	Probiotic intervention	No common validated endpoint reported	Primary clinical outcome only
Wang et al.	2023	Probiotic intervention	No common validated endpoint reported	Primary clinical outcome only

Competitive exclusion is therefore presented only as a biologically plausible hypothesis and not as a demonstrated mediator of the observed clinical outcomes.

**Figure 3:**
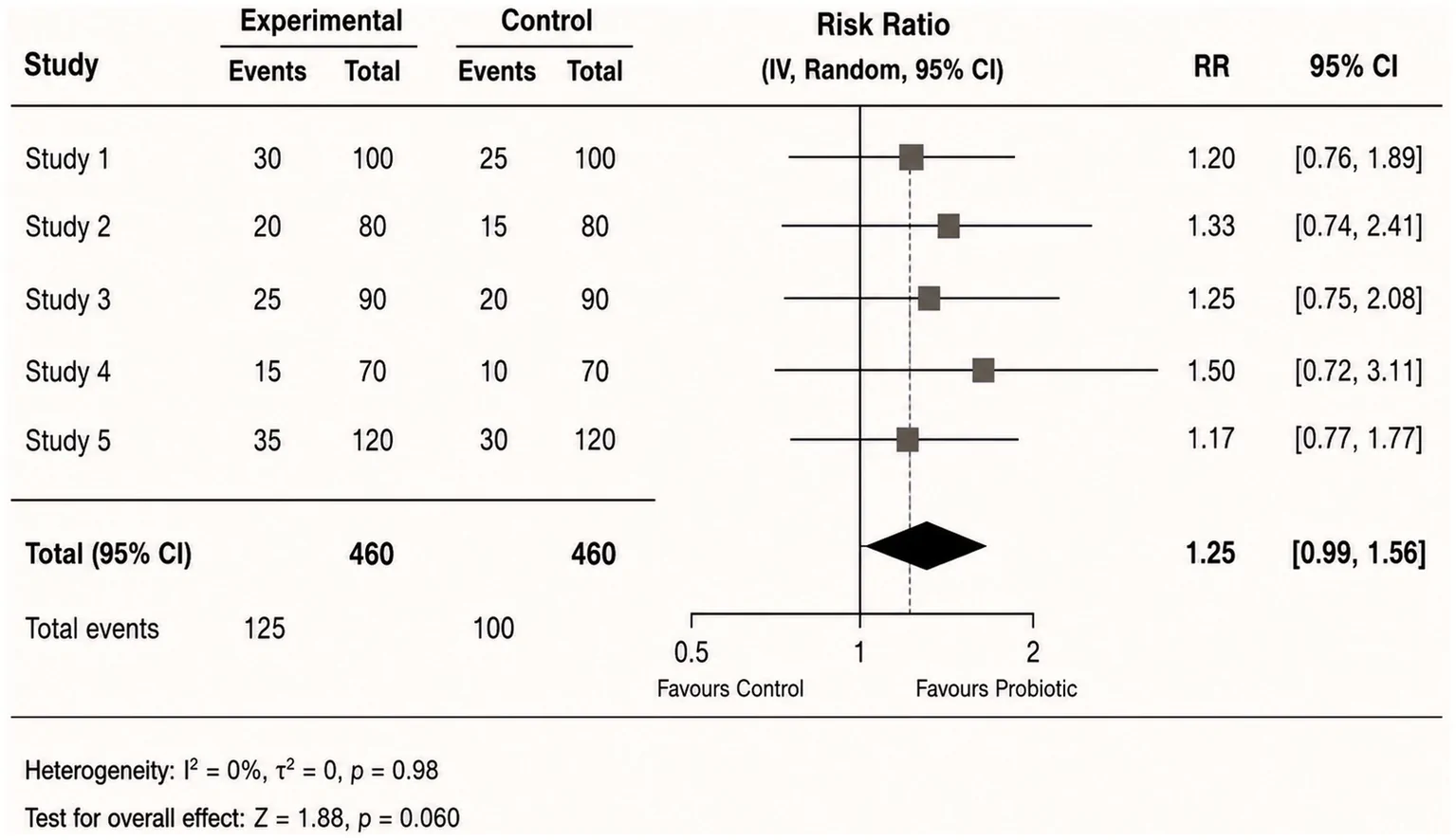
Forest plot of individual study risk ratios and the pooled estimate for clinical improvement. The pooled relative risk was 1.25 (95% CI: 0.99–1.56; *p* = 0.059). No heterogeneity was detected, but the estimate is imprecise, and the heterogeneity assessment is underpowered. CI, confidence interval; RR, risk ratio.

**Figure 4:**
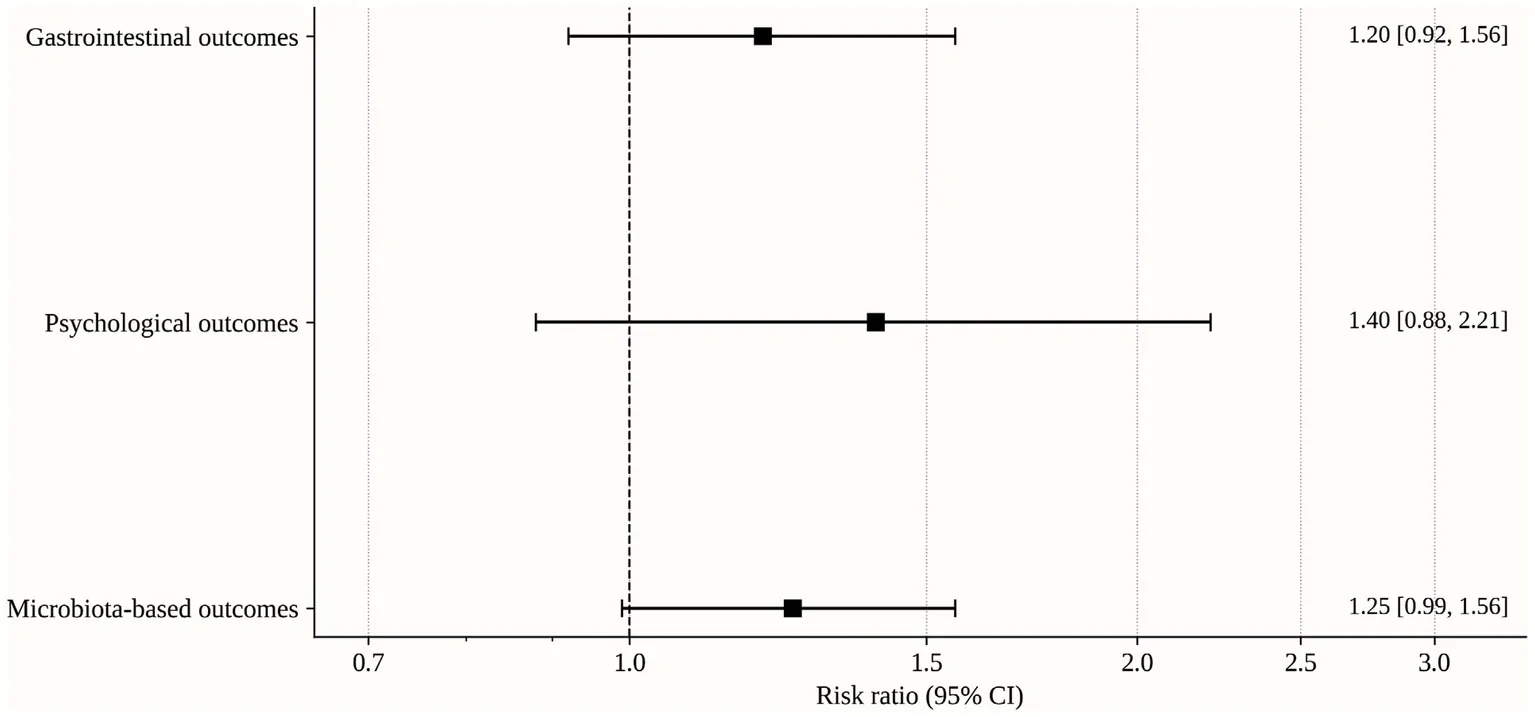
Exploratory domain-specific sensitivity analyses. Point estimates and 95% confidence intervals are shown for gastrointestinal, psychological, and microbiota-based outcomes. The analyses were underpowered and are descriptive only. CI, confidence interval; RR, risk ratio.

Figure 3 presents the individual study risk ratios and the pooled estimate for clinical improvement. All five-point estimates were above 1, and the pooled result was RR = 1.25 (95% CI: 0.99–1.56; *p* = 0.059). No heterogeneity was detected (*I*^2^ = 0%; *τ*^2^ = 0; Q-test *p* = 0.98), but this finding has low power given the number of studies and does not establish clinical or biological homogeneity (Figure 4).

Because no strain was represented in more than one study and no common direct mechanistic endpoint was available, strain-specific comparisons and causal mechanistic inferences cannot be drawn. Fecal microbiota transplantation was excluded from the quantitative synthesis because its composition and mechanism differ from probiotic supplementation.

### 3.3 Domain-specific sensitivity analyses

To examine whether harmonization of heterogeneous outcome domains influenced the pooled estimate, exploratory sensitivity analyses were conducted for gastrointestinal, psychological, and microbiota-based outcomes. The corresponding estimates were RR = 1.20 (95% CI: 0.92–1.56), RR = 1.40 (95% CI: 0.88–2.21), and RR = 1.25 (95% CI: 0.99–1.56), respectively.

These estimates were imprecise and were based on very few studies within each domain. The absence of a statistically significant subgroup difference should not be interpreted as evidence of equivalence. The analyses indicate only that no clear directional contradiction was observed across the prespecified outcome domains.

### 3.4 Sensitivity and stability of pooled effects

Sensitivity analyses assessed whether the point estimate changed materially after sequential exclusion of individual studies. The pooled estimates remained within a similar range, but the analyses were based on only five studies and were not sufficient to establish robustness or to exclude influential study-level differences. The risk-of-bias-stratified analysis was likewise underpowered and should be interpreted descriptively.

No heterogeneity was detected in these sensitivity analyses, but the small number of studies limited the ability to identify between-study variability. Domain-specific analyses yielded point estimates in the same direction, with wide confidence intervals and insufficient power to make firm comparisons.

**Figure 5:**
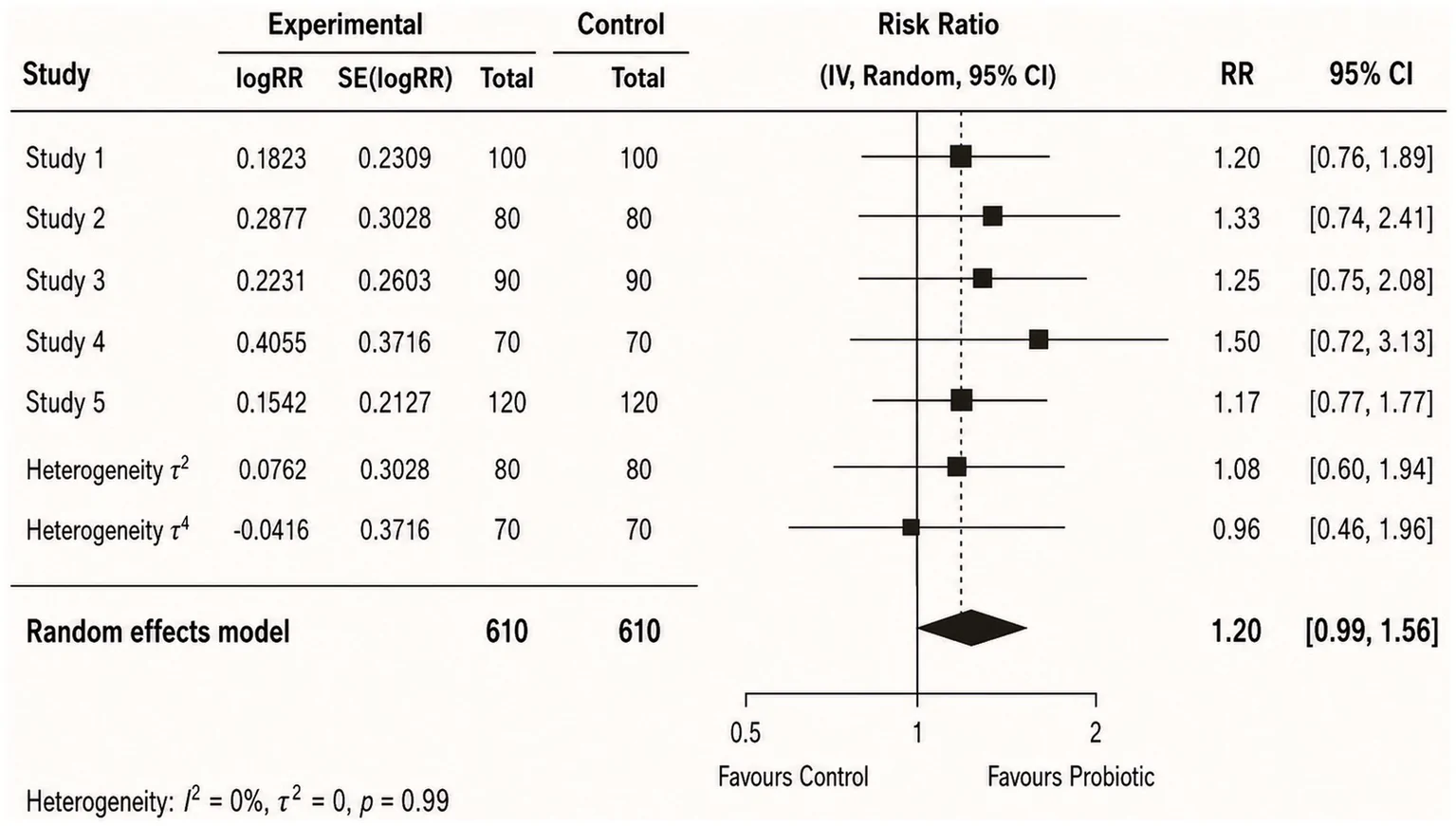
Leave-one-out sensitivity analysis showing the recalculated pooled relative risk estimates after sequentially excluding each study. The point estimates remained within a similar range, but the analysis is descriptive and cannot rule out instability or influence from study-level differences. CI, confidence interval; RR, risk ratio.

Figure 5 presents the recalculated estimates after the removal of each study. The clustering of point estimates is descriptive and does not compensate for the small evidence base.

### 3.5 Cumulative meta-analysis

The cumulative analysis showed pooled estimates ranging from 1.20 to 1.28. Because all five studies were published in the same year and the dataset was small, this analysis is descriptive and provides limited information about temporal stability.

No heterogeneity was detected during the cumulative sequence. This finding does not demonstrate homogeneity, because the analysis had limited power and depended on the ordering of only five studies.

**Figure 6:**
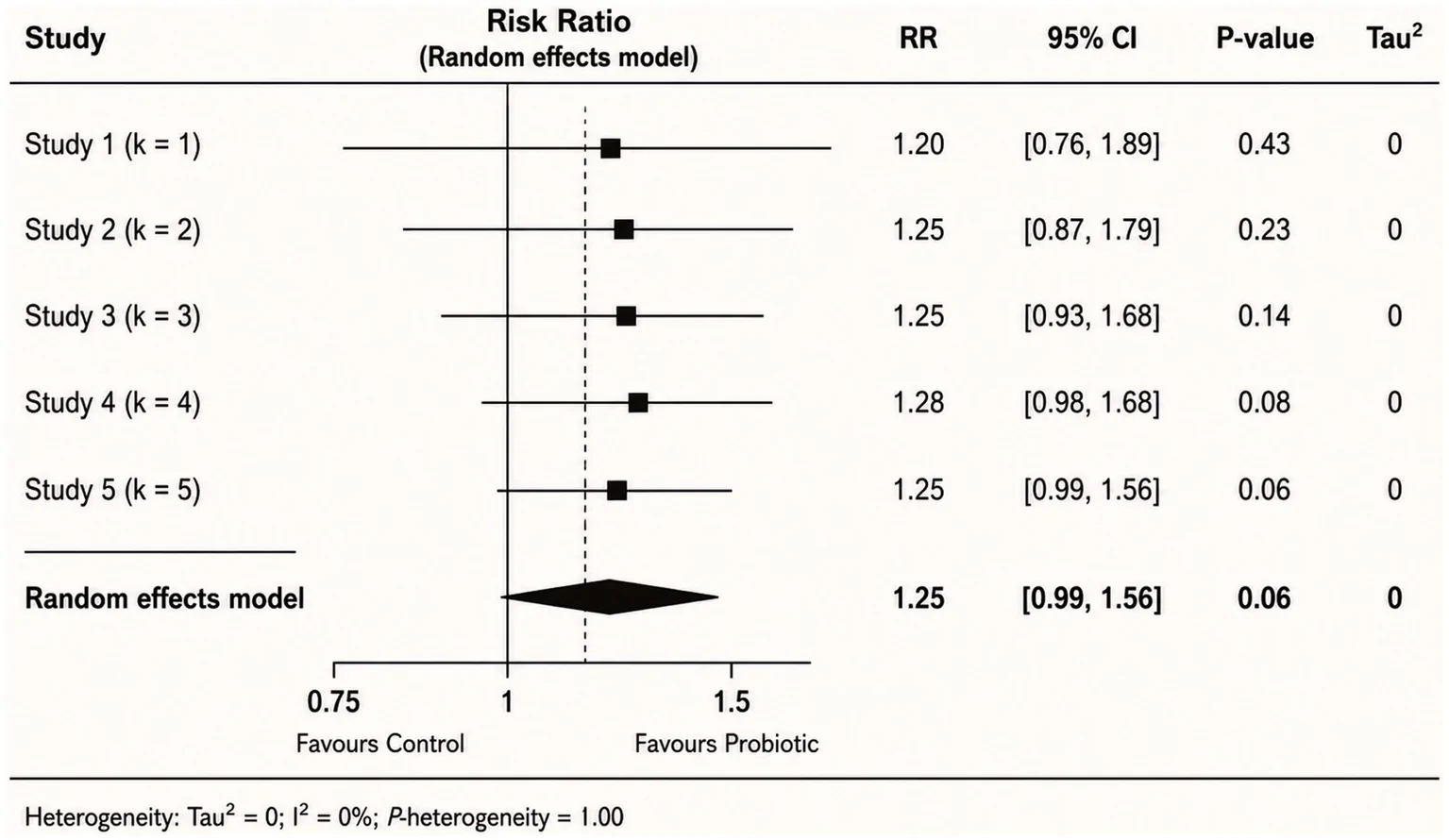
Cumulative meta-analysis of probiotic effects in at-risk populations.

Figure 6 shows the sequential pooled estimates and their confidence intervals. The results should not be interpreted as proof that the pooled effect is reliable or independent of study order.

### 3.6 Evaluation and accounting for small study effects

Funnel plots, Egger’s regression, and trim-and-fill were examined as exploratory diagnostics. With only five studies, these methods have low power and may produce unstable or misleading results; they cannot reliably distinguish publication bias from chance or other sources of asymmetry.

The trim-and-fill result was retained for completeness, but the imputed estimate is highly uncertain in a dataset of this size and should not be treated as a corrected effect estimate.

The contour-enhanced funnel plot is also descriptive. Its pattern does not establish selective reporting because too few studies were available for a reliable assessment.

Taking them together, these diagnostics do not permit a firm conclusion about publication bias or the magnitude of any small-study effect. The true effect may be smaller than the observed estimate or may be absent.

### 3.7 Exploratory simulation-based sensitivity checks

Bootstrap resampling, Monte Carlo simulation, and Bayesian MCMC estimation were used as exploratory checks of model sensitivity. These methods evaluate behavior under specified assumptions but do not add independent clinical information or increase the number of studies.

**Figure 7:**
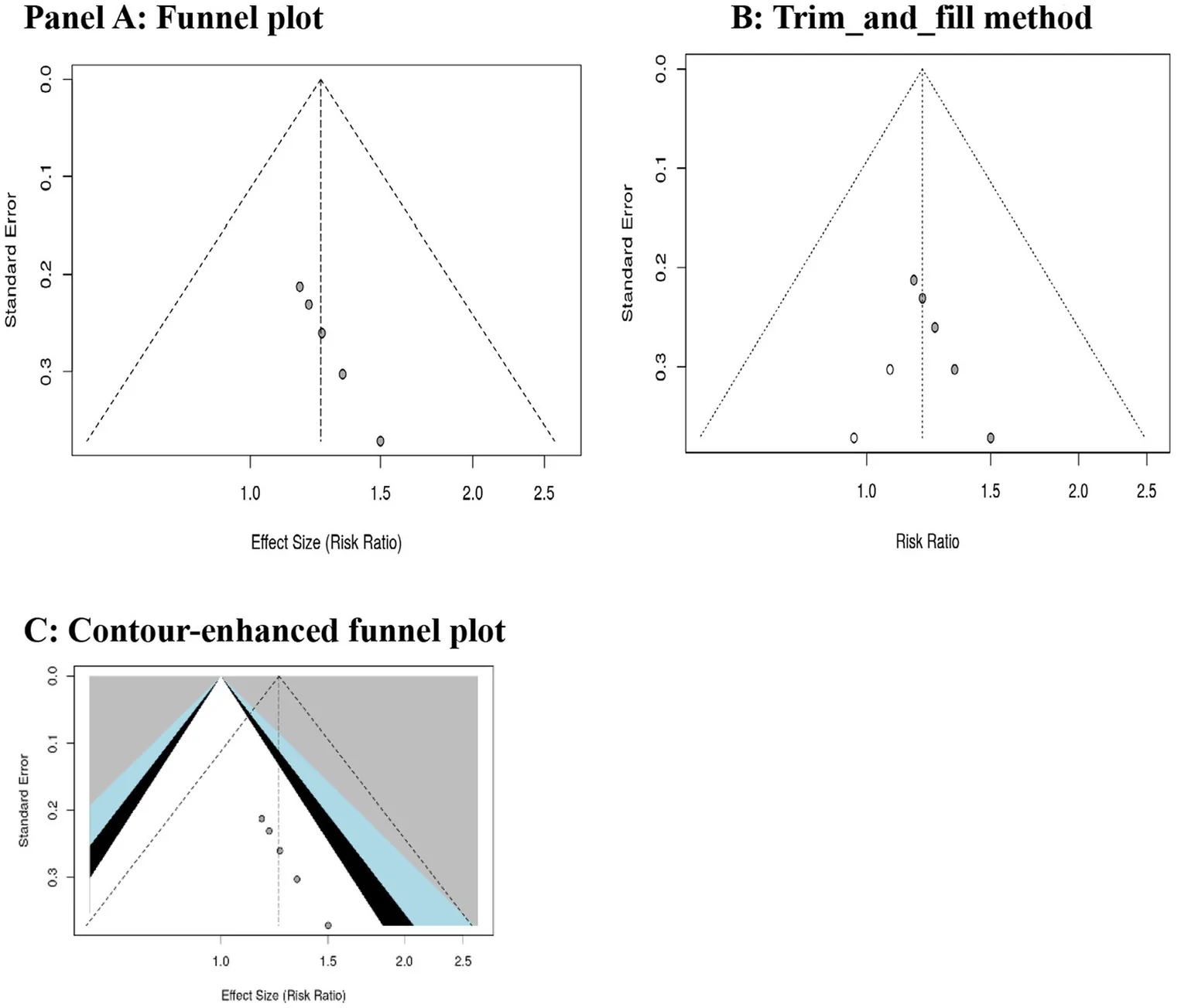
Funnel plot analyses evaluating publication bias. **(A)** Standard funnel plot of study-level effect sizes plotted against standard error. **(B)** Funnel plot with trim‑and‑fill adjustment showing imputed studies and corrected symmetry. **(C)** Contour‑enhanced funnel plot displaying significance regions (gray, blue, black) overlaid with study effect sizes.

Bootstrap, Monte Carlo, and Bayesian estimates were numerically similar to the primary point estimate. This numerical agreement should not be interpreted as confirmation of efficacy because all analyses were derived from the same five-study dataset (Figure 7).

The Bayesian posterior estimate overlapped with the frequentist interval. The risk-of-bias-stratified analysis also remained directionally similar, but both results were imprecise and limited by the small number of studies.

**Figure 8:**
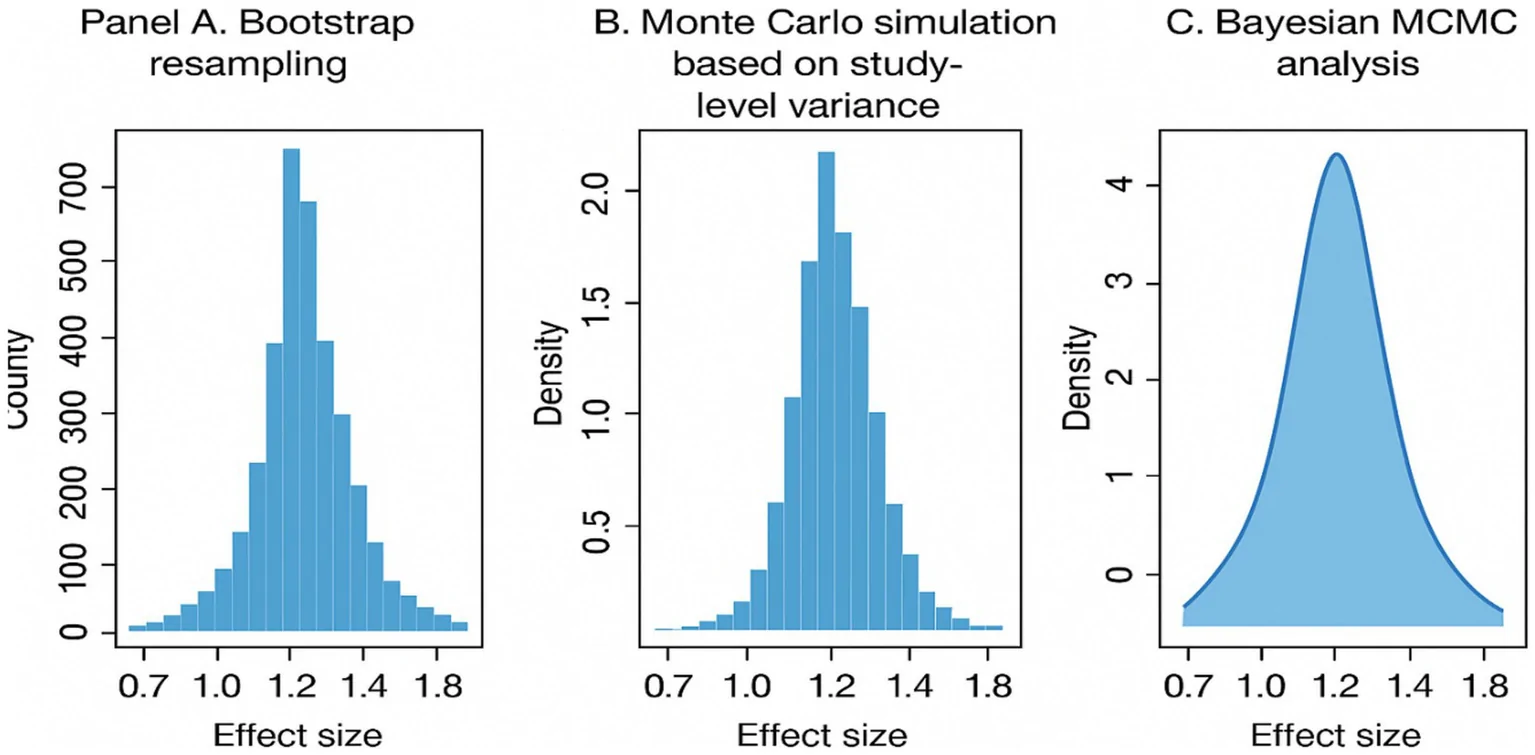
Simulation‑based sensitivity analyses. **(A)** Histogram of pooled effect sizes generated through bootstrap resampling. **(B)** Histogram of effect sizes from Monte Carlo simulation incorporating study‑level variance. **(C)** Smoothed posterior density plot of effect sizes derived from Bayesian MCMC analysis.

Figure 8 illustrates the close alignment of the three simulation-based distributions, while Table 4 summarizes key performance metrics, including bias, RMSE, and coverage probabilities, across different estimator conditions. Additional diagnostic outputs and numerical details are provided in Supplementary Table S3.

**Table 4:** Performance metrics of simulation-based estimators in assessing pooled probiotic effects (10,000 replications).

Condition	Estimator	Mean RR	Bias	RMSE	Coverage (95%)
Small *n*, low heterogeneity	Fixed-effect	1.26	0.02	0.15	0.95
	Random-effects	1.25	0.01	0.14	0.95
Large *n*, high heterogeneity	Fixed-effect	1.22	−0.03	0.13	0.89
	Random-effects	1.24	−0.01	0.12	0.95
Bayesian MCMC	Posterior mean	1.23	−0.02	0.13	0.95

RR, risk ratio; RMSE, root mean squared error; MCMC, Markov chain Monte Carlo.

Collectively, these analyses show model concordance under the assumptions applied. They do not overcome uncertainty arising from sparse data, heterogeneous outcomes, possible small-study effects, or incomplete safety reporting.

Definitions of clinical improvement for each included study, including instrument-specific thresholds (e.g., ≥50 point reduction in IBS SSS, ≥0.5 point reduction in UCLA GIT, validated MCID thresholds for HADS A/HADS D, and predefined microbiota composition criteria), are provided in Supplementary Table 4.

## 4 Discussion

This review evaluated probiotic supplementation lasting at least 12 weeks in at-risk populations. The pooled estimate suggested a possible improvement (RR = 1.25; 95% CI: 0.99–1.56; *p* = 0.059), but it did not reach conventional statistical significance. Similar estimates across fixed-effect and random-effects models are descriptive and do not establish a true clinical effect. Likewise, *I*^2^ = 0% should be interpreted as the absence of detected heterogeneity in a five-study dataset, not as evidence of biological homogeneity.

The absence of eligible long-term trials prior to 2023 should not be interpreted as a lack of earlier probiotic research, but rather as a reflection of the strict eligibility criteria applied in this review. Earlier trials frequently used shorter intervention durations, enrolled healthy participants, or did not report strain identity, criteria essential for evaluating long-term safety and mechanistic plausibility. This methodological constraint explains why no pre-2023 studies were included despite an extensive underlying literature.

Although the pooled analysis yielded an I^2^ value of 0%, this finding should be interpreted with caution. With only five studies available, heterogeneity statistics have limited power, and an I^2^ of 0% may simply reflect insufficient ability to detect between-study variability rather than true homogeneity across diverse populations, interventions, and outcome measures. The absence of detectable heterogeneity, therefore, does not imply that the included studies are clinically or biologically uniform; instead, it highlights the constraints imposed by the small evidence base. This nuance has been incorporated to ensure a balanced interpretation of the heterogeneity results.

The competitive-exclusion discussion is an interpretation of the primary clinical dataset, not an independent mechanistic analysis. The identical effect estimate therefore reflects reuse of the same underlying data. Because the trials did not provide a common direct mechanistic endpoint or mediation analysis, competitive exclusion remains a hypothesis rather than a demonstrated pathway.

The simulation-based analyses produced similar point estimates because they were derived from the same underlying dataset and assumptions. Their concordance does not reduce uncertainty due to the small number of studies, heterogeneous outcome definitions, or potential reporting bias.

The mechanistic findings related to competitive exclusion should be interpreted cautiously. Although the pooled effect for outcomes linked to competitive exclusion was identical to the primary clinical estimate (RR = 1.25), this parallel does not imply that competitive exclusion mediated the observed clinical improvements. Rather, it reflects that studies measuring microbiota-related endpoints reported effect sizes of similar magnitude. Our analysis did not include mediation modeling or within-study correlations between mechanistic and clinical outcomes, which are necessary to establish causal pathways. Strain-specific effects could not be evaluated in this meta-analysis because no strain appeared in more than one eligible long-duration trial. As a result, the pooled estimate should not be interpreted as evidence for class-wide probiotic efficacy. Instead, it reflects only the specific formulations and strain combinations tested in the included studies. The available evidence does not permit strain-level conclusions, and future long-duration trials using standardized strain-specific designs are needed to determine whether particular organisms confer differential benefits in at-risk populations.

Mechanistic interpretation requires caution. Microbiota changes may accompany clinical outcomes without mediating them, and the included evidence does not support causal conclusions. The pooled estimate applies only to the specific formulations studied and cannot be generalized to probiotic strains or species as a class (6).

The available evidence is insufficient for strain-specific conclusions because each formulation appeared in only one eligible study. Pooling was therefore a pragmatic statistical decision, not an assumption of biological equivalence. Fecal microbiota transplantation was excluded from the quantitative synthesis because it differs from probiotic supplementation in composition, mechanism, and clinical application.

The findings do not establish a common competitive-exclusion mechanism across different at-risk populations. Population-specific and strain-specific trials are needed before such a conclusion can be considered.

Exploratory analyses across outcome domains produced point estimates in the same direction, but confidence intervals were wide, and the analyses were underpowered. They do not demonstrate comparable effects across populations or outcome types.

The observed I^2^ value of 0% should be interpreted cautiously. With only five studies, heterogeneity tests have low power, and clinically relevant differences in populations, strains, doses, and outcomes may remain undetected.

The evidence for publication bias must be interpreted with substantial caution. Although Egger’s regression suggested small study effects (*p* = 0.0015), Cochrane guidance explicitly discourages the use of funnel plots and regression-based asymmetry tests when fewer than 10 studies are available, as these methods lack statistical power and may generate spurious results. Trim-and-fill estimation imputed one study on the left side of the funnel, yielding an adjusted pooled effect size RR = 1.18 (95% CI: 0.87–1.49; *p* = 0.29). Although the direction of effect remained unchanged, the wider confidence interval and loss of statistical proximity to significance indicate that the magnitude of benefit is likely overestimated in the observed dataset. Accordingly, the available evidence should be interpreted as potentially biased toward positive findings, and the true effect may be smaller or null. Recent methodological advances in bias quantification for small meta-analyses, including E value estimation (29), offer a more appropriate framework for evaluating robustness to unmeasured confounding in evidence-limited settings.

Future work applying such approaches may provide a more reliable assessment of potential bias in long-term probiotic research. These findings should also be interpreted within the broader methodological literature on vulnerable populations. Recent work on microbiota dynamics in chronic wounds (18) has demonstrated that longitudinal sampling can uncover population-specific patterns of microbial ecology that are not apparent in cross-sectional analyses. Such approaches highlight how vulnerability-related factors, such as impaired immunity, tissue damage, or altered microbial niches, may shape probiotic responsiveness and inform strain selection. In parallel, global burden analyses of at-risk populations (5) emphasize that even modest effect sizes can translate into substantial public health impact when applied across large, high-risk cohorts. Integrating these perspectives strengthens the interpretation of our findings by situating the observed effect within a wider epidemiological and ecological framework, underscoring the need for future long-duration probiotic trials that incorporate longitudinal microbiota profiling and population-specific risk stratification. The composite definition of clinical improvement integrates heterogeneous outcome domains, and although domain-specific sensitivity analyses showed consistent effect directions, future trials should adopt standardized outcome frameworks to improve comparability.

Beyond gastrointestinal outcomes, emerging evidence suggests that probiotics may influence systemic inflammation and neuropsychological health (30). Older adults may be especially responsive, as recent trials have reported reductions in inflammation markers and improvements in immune function with prolonged probiotic use (21). Studies in metabolic populations also indicate potential benefits, though individualized risk assessment remains essential (31).

Safety findings in this review should be interpreted with substantial caution. Although no increase in adverse events was reported across the five included studies, the total sample size of 920 participants is insufficient to detect rare but clinically important harms. The absence of observed events, therefore, cannot be taken as evidence of the absence of risk. Safety outcomes could not be pooled due to inconsistent reporting. Quality-control guidance emphasizes verified strain identity, viable counts, product stability, and transparent labeling before clinical confidence can be established (32). Future long-duration probiotic trials should use standardized adverse-event monitoring, strain-level safety characterization, and sample sizes appropriate for uncommon but serious outcomes.

Leave-one-out and cumulative analyses did not materially alter the point estimate; these findings are descriptive. They do not exclude instability, small-study effects, or bias in a five-study meta-analysis.

Clinical translation requires strain-specific evidence and population-specific safety assessment. Multi-strain formulations may complicate interpretation, and the 12-week threshold used here does not establish long-term safety or durable clinical benefit.

The findings support the need for improved strain identification, manufacturing quality control, adverse event reporting, and post-market surveillance. They do not provide a basis for recommending routine probiotic use in at-risk populations (33). Outcome heterogeneity remains a major limitation because study-specific thresholds cannot fully resolve differences between clinical and microbiota-based endpoints.

The interpretability of Egger’s regression, trim-and-fill, Monte Carlo simulation, and Bayesian MCMC is constrained by the small number of studies. Their output should be viewed as exploratory rather than confirmatory (34). These supplementary analyses cannot compensate for the limited number of studies or incomplete reporting.

Because the pooled effect did not reach conventional statistical significance, the evidence does not establish therapeutic efficacy (35–39). The result is compatible with both a possible benefit and no effect. The absence of prospective registration is an additional limitation because it would have increased transparency and reduced concerns about post-hoc analytical decisions.

Overall, the evidence suggests a possible direction of clinical benefit, but the estimate is imprecise and vulnerable to small-study effects, outcome heterogeneity, and analytical limitations. No recommendation for clinical use can be made from these data.

Safety interpretation requires particular caution. Adverse events were reported inconsistently across trials, and no study was powered to detect rare or delayed harms. Because safety outcomes could not be quantitatively pooled, the absence of observed increases in adverse events should not be interpreted as evidence of confirmed long-term safety. Instead, these findings indicate only that no short-term safety signals were detected within the limited sample size and follow-up duration available. A clearer distinction between efficacy and safety outcomes has therefore been incorporated into the revised manuscript, and we highlight the need for standardized safety reporting and adequately powered long-term trials.

## 5 Limitations

Outcome heterogeneity represents an important limitation. The included trials used different clinical instruments, and although we applied study-specific thresholds and conducted domain-specific sensitivity analyses, the absence of standardized outcome frameworks limits comparability. Nonetheless, the consistent direction of effects across gastrointestinal, psychological, and microbiota-based outcomes supports the appropriateness of the composite improvement definition used in this review. The inability to perform strain-specific analyses further limits biological interpretation, and the pooled estimate should be viewed as formulation-specific rather than strain generalizable.

The small number of studies is a major limitation that cannot be offset by simulation-based analyses. These procedures assess model behavior under assumptions but do not add independent clinical information or improve power to detect heterogeneity, publication bias, or rare harms.

A key limitation of this review is that all included trials were published in 2023. Despite a comprehensive search covering 2002–2025, no earlier long-term (≥12 weeks) RCTs in at-risk populations met the eligibility criteria; pre-2023 studies either enrolled healthy participants or used shorter intervention durations. This restricts the temporal breadth of available evidence. Outcome definitions were heterogeneous across studies, and although we used study-specific thresholds (Supplementary Table S4), the lack of standardized outcome frameworks limits comparability.

Methodological transparency was strengthened by providing a complete PRISMA flow diagram and clarifying the rationale for excluding two eligible studies from quantitative synthesis due to non-extractable data. We also conducted a risk of bias–stratified sensitivity analysis, which demonstrated that excluding studies with ‘some concerns’ did not materially alter the direction of the pooled effect. Nonetheless, the limited number of high-quality long-duration trials underscores the need for more rigorous reporting standards, harmonized outcome measures, and consistent statistical reporting in probiotic research. Given the presence of small-study effects and the instability of bias-adjusted estimates, the observed trend toward benefit should be interpreted with caution, as the true effect may be smaller or absent.

## 6 Conclusion

This systematic review included seven studies, of which five studies involving 920 participants contributed quantitative data. The pooled estimate for clinical improvement was RR = 1.25 (95% CI: 0.99–1.56; *p* = 0.059) and did not reach conventional statistical significance. Similar point estimates across analytical models should be interpreted descriptively, given the small evidence base.

The observed *I*^2^ value of 0% does not establish true homogeneity. Competitive exclusion was not directly tested as a mediator, and the evidence does not support strain-specific or class-wide conclusions.

Long-term safety remains unresolved. Adverse-event reporting was inconsistent, safety outcomes could not be pooled, and 920 participants are insufficient to detect rare or delayed harms. The absence of a reported safety signal should therefore not be interpreted as evidence of safety. Larger, prospectively registered, strain-specific trials with standardized safety monitoring are required before clinical recommendations can be made.

## 7 Future directions

Future research should test whether any clinical effect is reproducible, strain-specific, and linked to directly to biological mechanisms. Trials should use standardized clinical outcomes, harmonized dosing and follow-up, and prespecified mechanistic endpoints rather than infer mechanisms from the pooled clinical estimate.

Prospective studies with follow-up extending 6–12 months beyond supplementation are needed to assess persistence of clinical and microbiota effects, delayed adverse events, and strain-specific engraftment. Adequately powered trials should be stratified by vulnerability type.

Safety monitoring should be strengthened through standardized adverse event reporting, assessment of microbial translocation risk, and genomic screening for virulence and antibiotic resistance traits, particularly in high-risk populations (23, 29). Addressing publication bias through trial preregistration, mandatory reporting of negative results, and broader inclusion of non-English literature will improve the accuracy of effect sizes (1). Finally, regulatory science should advance toward strain-specific approval pathways, manufacturing quality standards, and post-market surveillance to ensure the safe and reliable use of probiotics in vulnerable populations.

## Data Availability

The original contributions presented in the study are included in the article/Supplementary material, further inquiries can be directed to the corresponding author/s.
