# Supplementary material for: Long-term safety and clinical effects of probiotic supplementation in at-risk populations: a systematic review and meta-analysis
Source: Front Nutr. 2026 Aug 18;13:1895136. doi: 10.3389/fnut.2026.1895136 (PMC13529487; doi:10.3389/fnut.2026.1895136)
Supplement: Supplementary file 2 [file Table_2.docx]

|  | Aithor and Year | Population Type  Gender and age | Study  Design | Duration intervention | Sample Size | Probiotic Species | Patients Disease | Dosage (CFU) | Group size  (control) and Event (control) | Primary outcome | Secondary Outcom | The statistical methods used to analyze the data in the included studies | Adverse Events Reported | Follow-up Duration | Effect Size (Cohen’s d) | p-value | Heterogeneity (I²) |
| --- | --- | --- | --- | --- | --- | --- | --- | --- | --- | --- | --- | --- | --- | --- | --- | --- | --- |
| 1 | Viet Dao,  et al.2020 | A total of 83 patients were included in the analysis, with a mean age of 43.9 years, and a majority (73.5%) of the participants were female. | - The study was a single-center uncontrolled trial conducted in Vietnam from January 2019 to January 2020 | - The duration of the study for patients using the multispecies probiotics product was 2 months, which is equivalent to approximately 60 days.  - Patients were followed up after 1 month and 2 months of using the probiotics, indicating that the total duration of the intervention and follow-up was 60 days. | A total of 202 patients participated in the anxiety-depression screening at Hoang Long clinic between January 2019 and January 2020. Out of these, 111 patients were eligible and received the multispecies probiotics product for the study. | Bifidobacterium bifidum W23, Bifidobacterium lactis W52, Lactobacillus acidophilus W37, Lactobacillus brevis W63, Lactobacillus | - The study focused on patients with chronic gastrointestinal (GI) symptoms who also exhibited symptoms of anxiety and depression. | The dosage form of the multispecies probiotic product is a dry powder supplied in sachets, with each sachet containing 2 grams of the product. Patients are instructed to mix one sachet in 100 mL of water, let it stand for 1 minute, and stir well before use. | there was no control group to compare the effects of the probiotics against a placebo or alternative treatment. | - The primary outcome of the study was the improvement in anxiety and depression symptoms, as measured by the Hospital Anxiety and Depression Scale (HADS) scores. | - The secondary outcome of the study included the assessment of gastrointestinal (GI) symptoms, which were evaluated through follow-up phone interviews after 1 and 2 months of using the multispecies probiotics product. The proportion of symptomatic patients decreased significantly over time, indicating an improvement in GI symptoms alongside the primary focus on anxiety and depression.  - Quality of life was also measured as a secondary outcome using the European Quality of Life scale (EQ-5D-5L). The results showed significant improvement in the EQ-5D-5L scores after 1 month and continued improvement after 2 months, suggesting that the use of the probiotics not only alleviated anxiety and depression symptoms but also enhanced the overall quality of life for the patients. | -The Cochran's Q test was utilized to evaluate changes in participants' symptoms between baseline, 1 month, and 2 months of using the multispecies probiotics product, allowing for the assessment of categorical data regarding symptom improvement. - Repeated-measures analysis of variance (RM-ANOVA) was employed to compare the differences in the Hospital Anxiety and Depression Scale (HADS) and European Quality of Life scale (EQ-5D-5L) scores over the study period, with Mauchly's sphericity test performed to check the assumption of sphericity, and Greenhouse-Geisser correction applied if necessary. | - A small proportion of patients (<5%) developed mild symptoms while using the multispecies probiotics product, including fullness, diarrhea, and sleep complaints. These symptoms were generally mild and did not lead to significant adverse effects. - One patient experienced a burning sensation in the throat after one week of probiotic supplementation, which caused her to discontinue the product after the first month; however, this sensation disappeared after stopping the probiotics. | -Patients were followed up after 1 month and 2 months of using the multispecies probiotics product, with follow-up conducted via phone interviews to assess changes in anxiety and depression symptoms, quality of life, and any new symptoms developed during the treatment period. - The analytic population included only those patients who completed the 2-month follow-up, ensuring that the data collected reflected the outcomes after the full duration of the probiotic supplementation. | -The study reported significant improvements in anxiety and depression symptoms as measured by the Hospital Anxiety and Depression Scale (HADS) scores, with mean scores decreasing from 20.0 at baseline to 7.2 at 1 month and further to 4.9 at 2 months, indicating a substantial effect of the multispecies probiotics on these symptoms. - Although the study did not explicitly calculate effect sizes such as Cohen's d, the statistically significant differences in HADS scores (p < 0.001) suggest a strong positive effect of the probiotics on reducing anxiety and depression symptoms in patients with chronic gastrointestinal disorders. | -The p-value was considered statistically significant when it was less than 0.05 in the study, indicating that the observed changes in symptoms and scores were unlikely to have occurred by chance. - All differences in the Hospital Anxiety and Depression Scale (HADS) and European Quality of Life scale (EQ-5D-5L) scores after 1 month and 2 months of using the multispecies probiotics were statistically significant, with p-values reported as less than 0.001. | - The study discusses the heterogeneity in findings related to the effectiveness of probiotics on anxiety and depression symptoms, which may be attributed to differences in probiotic strains, dosages, and treatment durations across various clinical trials. This variability can lead to inconsistent results when comparing the efficacy of probiotics in different studies. - A meta-analysis that pooled results from 10 clinical trials indicated no significant difference between patients who used probiotics and those who received a placebo. This lack of significant findings may also reflect the heterogeneity in the methodologies and populations studied, underscoring the need for more clinical trials with consistent probiotic strains, dosages, and treatment durations to better understand their effects. |
| 2 | Adrian Moser, 2018 | The study included ten patients diagnosed with diarrhea-predominant irritable bowel syndrome (IBS-D), who were symptomatic according to current S3 guidelines and aged between 18 and 65 years. | - The study was designed as a pilot study | -Patients recorded the oral administration of the synbiotic mixture twice a day for the entire 4-week period. | The study involved a sample size of ten patients diagnosed with diarrhea-predominant irritable bowel syndrome (IBS-D) who underwent a 4-week oral synbiotic treatment. | - The synbiotic treatment used in the study contained multiple species of probiotic strains, but specific strains were not identified as being able to colonize the gut. | - The study focused on patients with chronic gastrointestinal (GI) symptoms who also exhibited symptoms of anxiety and depression. |  | There was no control group mentioned in the study, | - The primary outcome of the study was the assessment of disease severity in patients with diarrhea-predominant irritable bowel syndrome (IBS-D), measured using the IBS Severity Scoring System (IBS-SSS). | - The treatment with the oral synbiotic led to a significant reduction in fecal zonulin concentration, indicating an improvement in intestinal barrier function, with values decreasing from 67 ng/ml to 36 ng/ml (p = 0.035). This suggests that the synbiotic may help in restoring the integrity of the gut barrier in IBS-D patients.  - There was a notable decrease in disease severity as measured by the IBS-SSS (Irritable Bowel Syndrome Severity Scoring System), with scores dropping from 237 to 54 (p = 0.002) after the 4-week synbiotic treatment, indicating an improvement in overall symptoms and quality of life for the patients | - Statistical analyses were carried out using SPSS 22 (IBM Corporation, USA) and GraphPad Prism 6.0 (GraphPad Software, Inc., USA) to evaluate the data collected from the study participants before and after the synbiotic treatment. - The study employed various statistical tests to determine significance, including comparisons of microbial diversity, immune cell percentages, short-chain fatty acid levels, and zonulin concentrations, with p-values reported for each parameter to indicate the level of statistical significance. | - The study does not report any adverse events associated with the 4-week oral synbiotic treatment in the ten patients with diarrhea-predominant irritable bowel syndrome (IBS-D). All patients continued their usual medications without any new medications or changes in dosage during the study period. - The inclusion and exclusion criteria ensured that patients with chronic inflammatory diseases, immune disorders, or those on immune-modifying medications were ruled out, which may have contributed to the absence of reported adverse events during the trial. | - The follow-up duration for the study was 4 weeks, during which patients recorded the oral administration of the synbiotic mixture twice a day. - At the end of the 4-week period, all examinations were re-performed, including endoscopy and obtaining of fecal samples, to assess the effects of the synbiotic treatment. | - The study did not provide specific calculations or values for Effect Size (Cohen’s d) in relation to the outcomes observed after the 4-week oral synbiotic treatment in IBS-D patients. Instead, it focused on reporting p-values for various comparisons, indicating statistical significance without detailing effect sizes. - The findings highlighted significant changes in mucosal microbiota diversity, immune cell composition, and clinical symptoms (measured by IBS-SSS), but the absence of Effect Size calculations limits the ability to quantify the magnitude of these changes in a standardized manner. | - The study reported several p-values indicating the statistical significance of the findings. For example, the increase in microbial diversity in gastric and duodenal mucosal specimens after synbiotic treatment had p-values of 0.008 and 0.025, respectively, suggesting a significant effect of the treatment on microbial diversity in these regions. - Additionally, the treatment resulted in significant changes in fecal short-chain fatty acids (SCFAs), with p-values of 0.007 for acetate and 0.037 for butyrate, indicating that the synbiotic treatment effectively elevated these important metabolites in the fecal samples of IBS-D patients. |  |
| 3 | Borja Ánchez, 2017 | Specific populations, such as bifidobacteria, are abundant in breast-fed infants and are generally considered indicators of good health, while aberrancies in gut microbiota composition are associated with various diseases, including chronic conditions like type 2 diabetes and inflammatory bowel disease. |  | - Probiotics have been shown to be effective in shortening the duration of infectious diarrhea, with clinical studies indicating positive and consistent results in reducing stool frequency and duration of symptoms. | The European Society for Paediatric Gastroenterology Hepatology and Nutrition (ESPGHAN) suggests that there is very low quality of evidence regarding the use of probiotics for acute gastroenteritis in children, indicating that more robust studies with larger sample sizes are needed to draw definitive conclusions about their efficacy. | - Probiotic species such as Lactobacillus GG and Saccharomyces boulardii have been shown to be effective in preventing antibiotic-associated diarrhea (AAD) and in the protection against Clostridium difficile-associated diarrhea (CDAD). These strains have been supported by clinical studies demonstrating their efficacy in alleviating symptoms related to these conditions. | - Inflammatory bowel disease (IBD) includes conditions such as Crohn's disease, ulcerative colitis (UC), and pouchitis, |  |  | - The primary outcome of probiotic dietary interventions is to achieve a positive effect on the health of the target population, which can be accomplished through various actions such as modulation of immunological responses, improvement of the nutritional state of the host, and modulation of the gut microbiota. |  | - Linear discriminant analysis is utilized to identify enzymatic pathways that show significant differences between pre- and post-intervention groups, helping to analyze the impact of dietary changes on gut microbiota and its functional pathways. - Systems biology methods and tools, such as Cytoscape, are employed for visualizing and analyzing interaction networks, enabling multiscale data integration, data clustering, enrichment analysis, and network comparison to understand the metabolism and modulation of intestinal microbiota. | - The text does not provide specific information regarding adverse events associated with probiotics. It primarily discusses the beneficial effects and mechanisms of probiotics, as well as their applications in various gastrointestinal conditions. - While the text mentions the need for safety and clinical studies to support the efficacy of probiotics, it does not detail any adverse events or negative side effects that have been reported in relation to probiotic use. |  |  |  |  |
| 4 | K Kajander, et al,2017 | Participants were aged between 20 and 65 years, and they were required to have undergone a colonoscopy or barium enema within the preceding 5 years, ensuring that they had no organic intestinal diseases or significant gastrointestinal surgery history. | - The study was a 5-month randomized double-blind, placebo-controlled, parallel-group intervention involving 86 patients diagnosed with irritable bowel syndrome according to the Rome II criteria. | - The study included a preceding 3-week washout period and a 3-week follow-up period, contributing to the overall timeline of the intervention. | The study initially aimed to have 47 patients per group based on earlier assumptions regarding the baseline-adjusted composite IBS symptom score, which would provide sufficient power to detect a statistically significant difference between the probiotic and placebo groups. | - The multispecies probiotic supplementation used in the study included four specific strains: Lactobacillus rhamnosus GG, L. rhamnosus Lc705, Propionibacterium freudenreichii ssp. shermanii JS, and Bifidobacterium animalis ssp. lactis Bb12. These strains were selected for their potential benefits in alleviating symptoms of irritable bowel syndrome (IBS).  - Each probiotic strain was included in a daily dose of 4.8 x 10^9 bacterial cells, delivered through a milk-based drink that contained a combination of these probiotics, aimed at improving intestinal microbiota stability and reducing IBS symptoms over the course of the 5-month intervention. | - The patients in the study were diagnosed with Irritable Bowel Syndrome (IBS) based on the Rome II criteria, | - The control group consisted of 43 subjects who received a placebo during the 5-month intervention period. This group was randomized alongside the probiotic group to assess the effects of the multispecies |  | - The primary outcome of the clinical trial was the composite irritable bowel syndrome (IBS) score, which showed a significant decrease of 14 points (95% CI: −19 to −9) from baseline in the multispecies probiotic group after 5 months, compared to a decrease of only 3 points (95% CI: −8 to 1) in the placebo group, with a p-value of 0.0083 indicating statistical significance. | - The secondary outcome measures included the weekly scores of each symptom related to irritable bowel syndrome (IBS), bowel habits, and health-related quality of life (HRQL), with possible ranges of 0-28 for symptom scores and 1-7 for each HRQL domain. These measures were analyzed using the intention-to-treat population and the last-observation-carried-forward method. | - The study utilized intention-to-treat analysis for questionnaire data, employing the last-observation-carried-forward method to handle missing data. This approach ensures that all randomized participants are included in the analysis based on their initial group assignment, maintaining the integrity of randomization. - For the analysis of faecal samples, a similarity index was calculated to assess the stability of the microbiota composition between baseline and intervention samples. The mean logarithmic similarity index was compared between the probiotic and placebo groups, with statistical significance determined using appropriate P-values to evaluate differences in microbiota stability. | - Ten of the 43 subjects in the probiotic group and 15 of the 43 subjects in the placebo group reported at least one adverse event, with most events being symptoms related to the gastrointestinal or respiratory tract. In the probiotic group, 62 gastrointestinal events were reported, while the placebo group had 65 such events. - Other adverse events reported in the probiotic group included an eye operation, an atherosclerotic finding in the carotid artery, an inflamed mole, cystitis, and tenosynovitis. In the placebo group, events included oral herpes, breathing difficulties, hyperthyroidism, backache, a foot operation, an inflamed operation wound, vaginitis, and a prophylactic treatment against intestinal worms. Four of the adverse gastrointestinal events were considered to have a possible connection with the study, all occurring in the placebo group. | - The study included a follow-up period of 3 weeks after the 5-month intervention, during which participants were monitored for any changes in their condition or symptoms. - During the follow-up, patients met with the study doctor once to assess the long-term effects of the multispecies probiotic supplementation on their irritable bowel syndrome symptoms and overall health-related quality of life. | - The study reported a significant decrease in the composite irritable bowel syndrome (IBS) score at 5 months, with a reduction of 14 points in the probiotic group compared to a 3-point reduction in the placebo group, indicating a clinically meaningful effect size. The p-value for this difference was 0.0083, suggesting that the effect of the multispecies probiotic supplementation was statistically significant. - The improvement in health-related quality of life (HRQL) for the bowel symptoms domain was also noted, with a median change from baseline to the end of the study of 0.62 points in the probiotic group versus 0.37 points in the placebo group, with a p-value of 0.045 for the difference between groups, further supporting the effectiveness of the probiotic intervention. | - The p-value for the difference in the composite irritable bowel syndrome score between the probiotic group and the placebo group at 5 months was 0.0083, indicating a statistically significant reduction in symptoms with probiotic supplementation compared to placebo. - The p-value for the difference in the quality of life improvement in the bowel symptoms domain was 0.045, suggesting that the probiotic treatment had a beneficial effect on this specific aspect of quality of life compared to the placebo group. |  |
| 5 | P Marteau,  at al,2002 | Specific populations mentioned in the studies include elderly subjects, patients receiving multiple antibiotics, children with acute diarrhoea, and individuals with chronic conditions such as ulcerative colitis and Crohn's disease. | - The studies included in the analysis utilized randomized controlled trial (RCT) designs, which are considered the best evidence due to their ability to minimize bias. Specific studies mentioned include a double-blind RCT involving thirty-two patients with Crohn's disease, and another RCT with fifty-five volunteers with Helicobacter pylori infection, among others. | - Gastroenteritis typically heals spontaneously within a few days, although the exact duration can vary depending on the individual and the severity of the condition | The sample sizes in the clinical trials varied significantly, with some studies involving as few as 28 subjects, such as the trial on Crohn's disease with E. coli Nissle 1917, while others included larger groups, such as the trial with 571 healthy children aged 1-6 years attending day care centers. This variability in sample size can impact the statistical power and generalizability of the study findings. | Probiotics consist of various species, primarily including bacteria such as Lactobacillus and Bifidobacterium, as well as yeast like Saccharomyces. Specific strains mentioned include Saccharomyces boulardii, | - Probiotics have shown good evidence for efficacy in the treatment and prevention of various gastrointestinal diseases, including antibiotic-associated disorders, gastroenteritis, acute diarrhoea, and lactose intolerance. Specific strains such as Saccharomyces boulardii, Lactobacillus rhamnosus GG, and Enterococcus faecium SF68 have been highlighted for their positive effects in these conditions.  - In the context of inflammatory bowel disease (IBD), three probiotics—VSL3, E. coli Nissle 1917, and S. boulardii—have been recommended based on recent randomized controlled trials. These probiotics have demonstrated a significant reduction in relapse rates for patients with Crohn's disease and ulcerative colitis, indicating their potential role in managing chronic intestinal inflammation. | The control group had a higher relapse rate, with 616 relapses compared to 116 in the probiotic group. |  |  | - Secondary outcomes in clinical trials often refer to additional effects or benefits that are measured alongside the primary outcome. In the context of probiotics, secondary outcomes may include the reduction in the duration of diarrhoea, improvement in gastrointestinal symptoms, or changes in the levels of specific gut bacteria, which can provide insights into the overall effectiveness of the probiotic treatment beyond the main focus of the study.  - In studies involving probiotics for conditions like Clostridium difficile infections, secondary outcomes could also encompass the assessment of recurrence rates of infections, the severity of symptoms, and the quality of life of patients, which help to evaluate the broader impact of the probiotic intervention on patient health and well-being. | - The best evidence for the efficacy of probiotics comes from randomized controlled trials (RCTs) which are designed to minimize bias. These trials provide a systematic approach to evaluate the effects of specific probiotic strains on gastrointestinal diseases, ensuring that the results are reliable and valid. - In the studies mentioned, statistical analyses often focused on comparing the outcomes between the probiotic and placebo groups, with some trials reporting significant differences in recurrence rates of infections or symptoms. However, some studies had methodological issues, such as non-compliance and subgroup analyses, which raised questions about the robustness of the statistical conclusions drawn from the data. | - The text does not provide specific information regarding adverse events associated with the use of probiotics. It primarily discusses the efficacy of various probiotic strains in treating gastrointestinal disorders without detailing any negative side effects or adverse reactions reported in clinical trials. - While some studies mentioned the tolerance to treatment being better in the probiotic group compared to placebo, there is no comprehensive data on adverse events or side effects experienced by participants in the trials discussed. | - The follow-up duration in a recent European randomized controlled trial involving 287 children with acute diarrhoea was significant, with the study reporting a follow-up period during which the duration of diarrhoea was measured, specifically noting that the probiotic L. rhamnosus GG shortened the duration of diarrhoea from 77.42 hours to 56.17 hours in children with rotavirus infection. - In another randomized controlled trial that included 204 undernourished Peruvian children, the follow-up duration was assessed to determine the effect of L. rhamnosus GG on the risk of diarrhoea, with the study indicating that the probiotic reduced the incidence of diarrhoea in non-breast-fed infants over the course of the year. |  | - The p-value is a statistical measure that helps determine the significance of results obtained in a study. It indicates the probability of observing the results, or something more extreme, if the null hypothesis is true. A lower p-value suggests that the observed data is less likely under the null hypothesis, often leading researchers to reject the null hypothesis in favor of an alternative hypothesis. - In the context of clinical trials, a p-value less than 0.05 is commonly considered statistically significant, meaning there is less than a 5% probability that the observed results are due to chance. This threshold is often used to assess the efficacy of treatments, such as the effectiveness of probiotics in preventing or treating gastrointestinal disorders, where specific p-values are reported to indicate the strength of the evidence supporting the treatment's effects. |  |
| 6 | Anna, Oniszczuk.,2021 | The text discusses the role of probiotics and prebiotics in alleviating various disorders, including those related to the immune system, cancer metastasis, type 2 diabetes, and obesity, but it specifically notes that little is known about their role in preventing or treating cardiovascular disease. |  |  |  | - While the text does not specify particular probiotic | - The text discusses the role of probiotics and prebiotics in alleviating various disorders, including those related to the immune system, cancer metastasis, type 2 diabetes, and obesity, |  |  | - The primary outcome of the review is to discuss the roles of gut microbiota, probiotics, and prebiotics in the prevention and treatment of cardiovascular disease, highlighting their potential as important dietary components. |  |  |  |  |  |  |  |
| 7 | Elisabet, Navarro,2020 | The population type discussed in the context of probiotic supplementation includes pregnant women, preterm neonates, and infants, highlighting the significance of gut microbiota establishment during the perinatal and infant period. |  |  |  | - Probiotic supplementation, particularly with mixtures of Lactobacillus and Bifidobacterium strains, | - Gut dysbiosis can lead to the development of various diseases, including atopic diseases, food sensitization, and infections such as necrotizing enterocolitis (NEC), |  |  | - The primary outcome of the review is to assess the effectiveness of probiotic supplementation in restoring gut microbiota balance during the perinatal and infant period, particularly in cases of gut dysbiosis caused by factors such as preterm delivery, formula feeding, and antibiotic treatment. |  |  | - The review discusses the safety of probiotic use in pregnant women, preterm neonates, and infants, indicating that probiotics can be a preventive or therapeutic tool for dysbiosis during the perinatal and infant period. - While the text does not explicitly mention specific adverse events reported, it implies a focus on the safety of probiotics in vulnerable populations, suggesting that any adverse events would be considered in the context of their therapeutic benefits. |  |  |  |  |
| 8 | Chencheng Xie,at al, 2019 | The population type studied in the context of probiotics and non-alcoholic fatty liver disease (NAFLD) includes both adults and pediatric patients, with clinical trials conducted over the past decade revealing mixed outcomes but overall suggesting a promising therapeutic strategy for NAFLD in these groups. | - The paper summarizes 26 randomized controlled trials (RCTs) that compared probiotics and/or synbiotics versus placebo in NAFLD patients from January 2010 to June 2019. | - The intervention duration for the study involving Manzhalii Bifidobacterium, Lactobacillus, Enterococcus, and Bacillus subtilis was for 1 month, which is approximately 30 days. | The text does not provide specific information regarding the sample size used in the studies or clinical trials related to probiotics | - The text does not specify particular probiotic species that may be beneficial for skin disorders, | - Non-alcoholic fatty liver disease (NAFLD) is characterized by an excessive accumulation of fat in the liver tissue, | - In the randomized controlled trial conducted by Malaguarnera et al., the control group consisted of 66 NASH patients, and the study aimed to evaluate the effects of probiotics on liver histology, specifically looking at hepatocyte ballooning, NAS score, and fibrosis after one year of treatment. |  | - The primary outcome of the randomized controlled trial conducted by Wong et al. with 20 NASH patients was a significant decrease in intrahepatic triglyceride content (IHTG) from a baseline of 22.6 to 14.9 in the probiotics group, while IHTG remained static in the usual care group. | - Secondary outcomes in clinical trials investigating probiotics and synbiotics for NAFLD often include various serum markers such as Gamma-glutamyl transferase (GGT), alkaline phosphatase (ALP), total cholesterol (TC), triglycerides (TG), low-density lipoprotein cholesterol (LDL-C), high-density lipoprotein cholesterol (HDL-C), and fasting serum glucose (FBS). These markers can show variability and may not directly reflect disease progression in the context of NAFLD. |  | - Although clinical trials rarely reported adverse effects or safety issues with probiotics in patients with NAFLD, there are a few case reports indicating potential adverse effects such as systemic infections (bacteremia and endocarditis), bowel ischemia, inflammatory reactions, D-lactic acidosis, and gastrointestinal side effects. - The Agency for Healthcare Research and Quality (AHRQ) concluded that existing probiotic randomized controlled trials (RCTs) reveal no evidence of increased risk; however, the lack of systematic reporting of adverse events in probiotic intervention studies limits the ability to clarify the safety of these interventions with confidence. | - The lack of long-term, post-intervention follow-up in clinical trials is a setback regarding the safety assessment of probiotics and synbiotics in patients with NAFLD. This absence of follow-up makes it difficult to evaluate the sustainability of the probiotic or synbiotic protective effects on NAFLD over time. - Although clinical trials rarely reported adverse effects or safety issues with patients suffering from NAFLD, the need for more comprehensive safety evaluations and long-term follow-up studies is emphasized to better understand the efficacy and safety profiles of probiotic and synbiotic interventions. |  |  | - Heterogeneity in the context of meta-analyses refers to the variability or differences in study outcomes that can arise from various factors such as differences in study populations, interventions, and methodologies. In the studies mentioned, analyses showed heterogeneity when evaluating the effects of probiotics/synbiotics on hepatic steatosis, indicating that the results may not be consistent across all studies included in the meta-analysis. - The presence of heterogeneity (I²) can impact the interpretation of meta-analytic results, as it suggests that the studies may not be measuring the same underlying effect or that there are other confounding variables influencing the outcomes. This necessitates careful consideration when drawing conclusions about the efficacy of probiotics/synbiotics in improving liver health in patients with non-alcoholic fatty liver disease (NAFLD). |
| 9 | Magdolna, Szántó., at al,2019 | The paper discusses common inflammatory skin diseases such as atopic dermatitis, psoriasis, rosacea, and acne vulgaris, which are associated with gut dysbiosis and may benefit from probiotic supplementation.  It also addresses less common but serious skin pathologies like erythema nodosum, pyoderma gangrenosum, and hidradenitis suppurativa, which could be linked to disturbances in gut flora composition. |  | - The trial involved a total duration of 120 days, with the first 60 days dedicated to administering either probiotics or placebo to the participants. | The text does not provide specific information regarding the sample size of studies related to probiotic supplementation and inflammatory skin disorders.  - It mentions that very few studies have investigated the influence of probiotic supplementation on inflammatory skin disorders, but does not detail the sample sizes used in those studies. | - The text does not specify particular probiotic species | - The paper discusses common inflammatory skin diseases such as atopic dermatitis, psoriasis, rosacea, and acne vulgaris, | - The control group consisted of 21 subjects who received a placebo during the trial. |  | - The primary outcome of the research is to explore the therapeutic potential of oral probiotics in managing inflammatory skin disorders, particularly focusing on conditions such as atopic dermatitis, psoriasis, rosacea, and acne vulgaris, which are associated with gut dysbiosis. |  |  | - The text does not provide specific information regarding adverse events reported in relation to probiotic supplementation for skin disorders. It primarily discusses the potential benefits and the need for more research in this area. - There is an acknowledgment of the heterogeneity in the supplemental regimens used in different studies, which may complicate the understanding of any adverse effects associated with probiotic use in the management of inflammatory skin diseases. |  |  |  | - The term "heterogeneity" in the context of the studies on probiotic supplementation for inflammatory skin disorders refers to the variability in the supplemental regimens applied across different research studies. This variability makes it challenging to draw consistent conclusions about the effectiveness of probiotics in managing skin conditions. - Due to this heterogeneity, the promising results observed in some studies regarding the influence of probiotics on inflammatory skin disorders are difficult to implement in clinical practice, as there is no standardized approach to probiotic supplementation that can be universally applied. |
| 10 | A.H.L., Low., at al,2018 | The population type in this study consisted of 40 subjects diagnosed with systemic sclerosis (SSc), specifically those experiencing gastrointestinal (GI) manifestations associated with the condition. | - The study is a double-blind randomised placebo-controlled trial conducted to assess the effects of probiotics on gastrointestinal symptoms in patients with systemic sclerosis (SSc), with a sample size of 40 subjects. | - The trial involved a total duration of 120 days, with the first 60 days dedicated to administering either probiotics or placebo to the participants | - The study involved a total of 40 subjects with systemic sclerosis (SSc), who were randomized into two groups: 21 subjects received a placebo and 19 subjects received probiotics. | - The probiotics used in the trial consisted of a composite of lactobacilli, bifidobacteria, and streptococcus, administered at a dosage of 900 billion units per day for 60 days. | - The patients in the study have systemic sclerosis (SSc), a chronic autoimmune disease that can lead to fibrosis and damage in various organs, including the gastrointestinal (GI) tract. | - The control group consisted of 21 subjects who received a placebo during the trial. |  | - The primary outcome of the trial was to assess the change in total Gastrointestinal Tract (GIT) scores between the probiotic and placebo groups after 60 days of treatment. The results showed that the change in total GIT was not statistically significant between the two groups, with the placebo group showing a change of −0.14±0.27 and the probiotic group | - At the secondary endpoint of the trial, there was a greater reduction in total GIT scores in the probiotic group (n=13; −0.18±0.26) compared to the initial placebo group (n=15; −0.05±0.22), although this difference did not reach statistical significance (p=0.14). | - Two-sample t-tests were employed to evaluate between-group differences in the total GIT change, with results reported as mean ± SD. This statistical method was used to compare the outcomes between the probiotic and placebo groups at both the primary and secondary endpoints. - An intention-to-treat analysis and last observer carried forward analysis were conducted to ensure that all randomized subjects were included in the final analysis, regardless of whether they completed the study or adhered to the treatment protocol. | - The majority of adverse events reported during the trial were classified as grades I and II, indicating that they were generally mild to moderate in severity. - The study did not report any serious adverse events, suggesting that probiotics were safe for use in patients with systemic sclerosis during the trial period. | - The trial involved a follow-up duration of 60 days for the primary endpoint assessment, where the change in total GIT was evaluated between the probiotic and placebo groups. - A secondary follow-up duration of 120 days was also conducted, which showed a greater reduction in total GIT in the probiotic group compared to the initial placebo group, although this difference did not reach statistical significance. | - The study did not report specific effect sizes such as Cohen's d for the primary or secondary endpoints, focusing instead on p-values to assess statistical significance between the probiotic and placebo groups. - The results indicated that while the primary endpoint showed no statistically significant difference in total GIT change between the groups (p=0.85), there was a trend towards greater improvement in the probiotic group at the secondary endpoint (p=0.14), particularly in the GIT-reflux subdomain where a significant reduction was observed (p=0.0037). | - The p-value for the primary endpoint, which assessed the change in total GIT between the placebo and probiotic groups after 60 days, was 0.85, indicating no statistically significant difference between the two groups. - For the secondary endpoint, the p-value was 0.14, suggesting that while there was a greater reduction in total GIT in the probiotic group compared to the initial placebo group after 120 days, this difference did not reach statistical significance. |  |
| 11 | Chujin Cao, at al,2023 | The population type primarily discussed in the context of non-alcoholic fatty liver disease (NAFLD) includes patients with obesity, type 2 diabetes mellitus (T2DM), and those with metabolic syndrome, as these groups are at an increased risk for developing NAFLD. | - The review summarizes various clinical trials on probiotics for NAFLD, but it does not provide specific details regarding the study design, publication year, country of origin, or sample size for each included study | - The duration of probiotic studies on NAFLD varies, with some clinical trials conducted over periods such as 8 weeks, 12 weeks, and 3 months, indicating a range of approximately 56 to 90 days for these interventions. | - The review does not specify the sample sizes of the clinical trials included in the analysis, indicating that the exact number of participants in each study is not provided in the summarized results.  - It mentions that randomized, double-blinded, and placebo-controlled trials were commonly undertaken, but does not detail the sample sizes or participant demographics for these studies. | Bifidobacterium and Lactobacillus being commonly referenced, although their effects can vary and are sometimes inconsistent across studies.  - Multi-probiotic formulations, such as the 'Symbiter' which contains a concentrated biomass of 14 probiotic bacteria genera including Bifidobacterium, Lactobacillus, Lactococcus, and Propionibacterium, have demonstrated benefits in reducing liver enzymes and improving lipid profiles in patients with NAFLD, suggesting that combinations of strains may be more effective than single strains. | - Non-alcoholic fatty liver disease (NAFLD) is a global public health issue with a prevalence of approximately 25% worldwide, | - The review discusses various clinical trials on probiotics for NAFLD, but specific details regarding the group size of control groups in these trials are not provided in the text. |  | - The primary outcome of the clinical trials on probiotics for NAFLD includes the improvement of liver parameters, specifically the reduction of liver enzymes such as alanine aminotransferase (ALT) and aspartate aminotransferase (AST), which are indicators of liver injury and function. | - The secondary outcomes of probiotics treatment in patients with NAFLD include the assessment of liver injury markers such as alanine aminotransferase (ALT), aspartate aminotransferase (AST), and gamma-glutamyl transferase (GGT). Clinical trials have shown that probiotics can lead to a reduction in these liver enzymes, indicating an improvement in liver function. |  | - The review does not provide specific information regarding adverse events reported in clinical trials involving probiotics for NAFLD, indicating a lack of detailed data on this aspect in the studies summarized. - While probiotics are generally considered safe, the review highlights that the effects and beneficial roles of probiotics in treating NAFLD are still in dispute, suggesting that further research is needed to fully understand their safety profile and any potential adverse effects. | - The duration of studies on probiotics for NAFLD varies, and it has been noted that both the duration of the study and the dosage of probiotics can influence the results of efficacy. Long-term studies are suggested to provide a higher level of evidence regarding the effects of probiotics on NAFLD. - Some clinical trials have reported follow-up durations ranging from 8 weeks to 3 months, with varying outcomes on liver parameters and lipid profiles, indicating that the length of follow-up may impact the observed benefits of probiotic treatment in patients with NAFLD. |  | - The text does not provide specific information regarding p-values or statistical significance related to the clinical trials of probiotics on non-alcoholic fatty liver disease (NAFLD). It discusses the effects of probiotics on liver parameters and metabolic profiles but does not mention any statistical analyses or p-values associated with these findings. - The review summarizes various clinical trials and their outcomes, indicating that while probiotics may improve certain liver functions and lipid profiles in patients with NAFLD, the exact statistical significance of these results, including p-values, is not detailed in the provided information. |  |
| 12 | Joseph, J.Y., Sung.,at al, 2023 | The population type studied in the systematic review and meta-analysis consists of patients diagnosed with non-alcoholic fatty liver disease (NAFLD). | - The systematic review included a total of 41 randomized controlled trials (RCTs) that focused on the treatment of non-alcoholic fatty liver disease (NAFLD) patients with probiotics, prebiotics, and synbiotics. The studies varied in design, with 18 involving probiotics, 17 involving synbiotics, and 6 involving prebiotics.  - The publication year, country of origin, and specific sample size for each included study were not provided in the abstract, indicating a lack of detailed information on these aspects within the available text. | - The text does not provide specific information regarding the duration of the interventions in the randomized controlled trials (RCTs) included in the systematic review and meta-analysis. | - A total of 41 randomized controlled trials (RCTs) were included in the systematic review, which comprised 18 studies focusing on probiotics, 17 on synbiotics, and 6 on prebiotics.  - The systematic review aimed to analyze the effects of these microbiome-targeted therapies on liver-related outcomes in patients with non-alcoholic fatty liver disease (NAFLD). | - The systematic review included a total of 41 randomized controlled trials (RCTs) that utilized various microbiome-targeted therapies, specifically 18 trials focused on probiotics, although the specific species of probiotics used in these trials were not detailed in the provided information.  - The findings indicated that these probiotic interventions significantly improved liver-related outcomes in patients with non-alcoholic fatty liver disease (NAFLD), including reductions in liver steatosis, fibrosis, and liver enzyme levels, but the heterogeneity in probiotic strains used across studies was noted as a limitation. | - Patients in the study are diagnosed with non-alcoholic fatty liver disease (NAFLD), |  |  | - The primary outcome of the systematic review and meta-analysis was the improvement in liver steatosis, fibrosis, and liver enzyme levels in patients with non-alcoholic fatty liver disease (NAFLD) following treatment with probiotics, prebiotics, and synbiotics. This was measured using ultrasound grading for liver steatosis and specific liver enzyme levels including alanine transaminase, aspartate transaminase, and gamma-glutamyl transferase. | - The systematic review and meta-analysis included a total of 41 randomized controlled trials (RCTs) that evaluated the effects of probiotics, prebiotics, and synbiotics on liver-related outcomes in patients with non-alcoholic fatty liver disease (NAFLD). The interventions significantly improved liver steatosis, fibrosis, and liver enzyme levels, indicating positive secondary outcomes related to liver health. | - The outcomes of the included studies were meta-analyzed using standardized mean difference (SMD) to quantify the effects of probiotics, prebiotics, and synbiotics on liver-related outcomes in NAFLD patients. - Study heterogeneity was assessed using Cochran's Q test and I2 statistics, which helped determine the variability in results across the different randomized controlled trials included in the systematic review. | - The systematic review and meta-analysis focused on the effects of probiotics, prebiotics, and synbiotics in improving liver-related outcomes in patients with non-alcoholic fatty liver disease (NAFLD), but it did not report any specific adverse events associated with these microbiome-targeted therapies. - Limitations in the existing literature, such as heterogeneity in probiotic strains, dosage, and formulation, were noted, but there was no mention of adverse events or side effects experienced by participants in the included randomized controlled trials. | - The abstract does not specify the follow-up duration for the randomized controlled trials (RCTs) included in the systematic review and meta-analysis of probiotics, prebiotics, and synbiotics in non-alcoholic fatty liver disease (NAFLD) patients. - As such, the follow-up duration remains unspecified, which may limit the understanding of the long-term effects of microbiome-targeted therapies on liver-related outcomes in NAFLD patients. | - The study utilized standardized mean difference (SMD) to analyze the effects of probiotics, prebiotics, and synbiotics on liver-related outcomes in patients with non-alcoholic fatty liver disease (NAFLD). The SMD values indicated significant improvements in various liver conditions, such as liver steatosis, fibrosis, and liver enzyme levels. - Specific SMD values reported include an improvement in liver steatosis with an SMD of 4.87, indicating a large effect size, and reductions in fibrosis and liver enzymes, with SMDs of -0.61 for fibrosis, -0.86 for alanine transaminase, -0.87 for aspartate transaminase, and -0.77 for gamma-glutamyl transferase, suggesting moderate to large effect sizes in these outcomes. | - The abstract does not provide specific p-values for the outcomes measured in the study, such as liver steatosis, fibrosis, or liver enzymes. Instead, it presents standardized mean differences (SMD) along with 95% confidence intervals (CIs) to indicate the effectiveness of microbiome-targeted therapies in improving liver-related outcomes in NAFLD patients. - The systematic review and meta-analysis highlight significant improvements in liver-related outcomes, but the absence of p-values suggests that the authors focused on effect sizes and confidence intervals to convey the results rather than traditional statistical significance measures. | - The study assessed heterogeneity among the included randomized controlled trials (RCTs) using Cochran's Q test and I² statistics, which are standard methods for evaluating the variability in study outcomes. - Despite the significant improvements observed in liver-related outcomes for NAFLD patients treated with microbiome-targeted therapies, the presence of heterogeneity in probiotic strains, dosage, and formulation among the studies was noted as a limitation that could affect the overall findings. |
| 13 | José García-Santos,  at al,2023 | The population type discussed in the paper primarily includes infants and children, particularly those suffering from gastrointestinal disorders (GIDs) and functional gastrointestinal disorders (FGIDs). These conditions are prevalent in the pediatric population and can have both short-term and long-term health consequences. | - The review highlights the need for well-designed and well-powered studies to identify specific gut microbiota dysbiosis patterns related to gastrointestinal disorders (GIDs) and to better understand the role of | days.    - For the management of acute infectious diarrhea, the ESPGHAN Working Group on Probiotics and Prebiotics guidelines suggest a duration of 5-7 days for probiotic strains such as Lacticaseibacillus rhamnosus GG and Saccharomyces boulardii. | The text mentions that many studies referenced regarding the efficacy of probiotics, particularly for conditions like irritable bowel syndrome (IBS) and functional gastrointestinal disorders (FGIDs), have limitations | - Probiotic species primarily include strains from the genera Lactobacillus and Bifidobacterium, as well as probiotic yeast such as Saccharomyces. | - Inflammatory bowel disease (IBD) is an immune-mediated condition that includes ulcerative colitis (UC), Crohn's disease (CD), and unclassified IBD (U-IBD), | does not provide specific details about the group size for control groups or the events associated with those control groups in the context of the trials |  | - The primary outcome of the studies reviewed focuses on the efficacy of probiotics in reducing the duration of diarrhea episodes, the number of stools per day, and the length of hospital stays in pediatric patients, particularly those infected with rotavirus. Probiotic treatments, especially with doses above 10^10 cfu/day, have shown to be more effective during the early stages of infection. |  | - The review highlights that the level of evidence from studies may be downgraded based on factors such as study quality, imprecision, and indirectness, which includes the mismatch of the study's population, intervention, comparison, and outcome (PICO) criteria with the question's PICO. This suggests a systematic approach to evaluating the robustness of the data analysis methods used in the included studies. - Additionally, the review indicates that a systematic review is considered to provide higher-quality evidence than an individual study, implying that statistical methods employed in systematic reviews may involve meta-analysis techniques to aggregate data from multiple studies, thereby enhancing the reliability of the findings. | - Adverse effects related to probiotic use were primarily reported in patients with compromised health, indicating that the majority of probiotic strains used are considered safe for the general pediatric population. The incidence of adverse events was similar whether a single strain or a mixture of probiotic strains was used. - Despite the established safety of probiotics in clinical trials, the long-term effects on human health remain unclear, necessitating further research to fully understand the implications of probiotic administration in various patient populations. | - The follow-up duration for studies on probiotics varies significantly, with many studies lacking standardized follow-up periods, which complicates the ability to draw consistent conclusions about the efficacy and safety of probiotic treatments in pediatric gastrointestinal diseases. - In the context of probiotic therapy for conditions such as necrotizing enterocolitis (NEC) and infant colic, the follow-up duration is often not clearly defined, leading to methodological limitations in the analyzed studies, including small populations and variability in treatment protocols. | - The text discusses the concept of effect size in the context of systematic reviews and individual studies, indicating that the level of evidence may be downgraded based on factors such as study quality, imprecision, and inconsistency between studies. A small absolute effect size may lead to a downgrade in the level of evidence, while a large or very large effect size could result in an upgrade. - The systematic review is considered to provide higher-quality evidence than an individual study, and the effect size is a critical factor in determining the strength of the evidence. The text emphasizes that the effect size should be interpreted cautiously, especially in studies with small sample sizes and varying probiotic strains, which can affect the overall conclusions regarding the efficacy of probiotics in treating gastrointestinal diseases in the pediatric population. |  |  |
| 14 | Håvard Fretheim, at al,2023 | The population type in the study consisted of patients with systemic sclerosis (SSc) who were experiencing moderate to severe lower gastrointestinal tract symptoms, specifically bloating and/or diarrhea, as assessed by the UCLA SCTC GIT score 2.0. | - The study was designed as a Norwegian multicenter, double-blind, randomized, placebo-controlled, phase 2 trial, assessing the safety and efficacy of fecal microbiota transplantation in patients with systemic | - The study followed patients in an open maintenance phase until week 20, which indicates a duration of approximately 140 days from the start of the trial (week 0) to the end of the maintenance phase (week 20). | - A total of 65 patients were randomized to receive either the standardized fecal microbiota culture (ACHIM) or a placebo in the study. |  | - Patients in the study have systemic sclerosis (SSc), a condition characterized by lower gastrointestinal tract (GIT) complications, | - A total of 65 patients were randomized to receive either the standardized fecal microbiota culture (ACHIM) or a placebo, with 33 patients in the ACHIM group and 34 patients in the placebo group. |  | - The primary outcome of the study was to assess the change between baseline and week 12 in the UCLA GIT score item for either diarrhea or bloating, depending on which symptom was the worst at baseline. This was evaluated separately for each patient and measured as the average marginal effect (AME). | - The secondary outcomes of the study included the assessment of safety and tolerability of the fecal microbiota transplantation (FMT) treatment, as well as the total UCLA GIT score, which measures the overall gastrointestinal symptoms experienced by the patients. | - The primary outcome was assessed by measuring the change in the UCLA GIT score item for diarrhea or bloating from baseline to week 12, evaluated separately for each patient, using the average marginal effect (AME) as the statistical measure. - Secondary outcomes included the total UCLA GIT score and the change in UCLA GIT score from week 12 to week 20, with statistical significance determined using p-values to compare the differences between the ACHIM and placebo groups. | - In the study, 16 patients (37%) in the ACHIM group and 19 patients (42%) in the placebo group experienced any side effects, which were generally mild and short-lasting. The most frequent side effect reported was abdominal pain, occurring in 5 patients (15%) in the ACHIM group and 2 patients (6%) in the placebo group. - One patient in the ACHIM group experienced an intramural perforation during gastroscopy, which required intravenous antibiotics; however, this patient fully recovered. The time to resolve abdominal pain was reported to be 2 days in both the ACHIM and placebo groups. | - The follow-up duration for the patients in the study was until week 20, which included an open maintenance phase after the initial treatment period. - Patients received an intestinal infusion of a standardized fecal microbiota culture (ACHIM) or placebo at weeks 0 and 2, followed by a second ACHIM infusion at week 12 before entering the maintenance phase. | - The study did not report a significant difference in the change in lower gastrointestinal (GI) symptoms between the ACHIM (fecal microbiota transplantation) and placebo groups, with an average marginal effect (AME) of 0.17 and a p-value of 0.25, indicating no meaningful effect size. - Similarly, there was no significant difference in the total GI score between the two groups over the same time period, with an AME of 0.09 and a p-value of 0.17, further suggesting that the effect size for fecal microbiota transplantation in improving lower GI symptoms in systemic sclerosis patients was negligible. | - The p-value for the change in lower gastrointestinal (GI) symptoms from week 0 to week 12 between the ACHIM and placebo groups was 0.25, indicating no significant difference in the improvement of symptoms between the two groups. - Similarly, the p-value for the total GI score change over the same time period was 0.17, which also suggests that there was no statistically significant difference in the overall GI symptoms between the ACHIM and placebo groups. |  |
| 15 | Kurvatteppa, Halemani., at al,2023 | The population type included in the systematic review and meta-analysis consisted of 946 pregnant women, 524 lactating mothers, and 1678 infants, indicating a focus on maternal and infant health. | - The systematic review and meta-analysis included sixteen trials with a total sample size of 946 pregnant women, 524 lactating mothers, and 1678 infants. | - The systematic review and meta-analysis included a total of 16 trials with a sample size of 946 pregnant women, 524 lactating mothers, and 1678 infants, but did not specify the duration of the probiotic interventions in days. | - The systematic review and meta-analysis included a total of 16 trials, which comprised 946 pregnant women, 524 lactating mothers, and 1678 infants. | - Probiotics were administered as interventions using either a single strain of Bifidobacterium or Lactobacillus, or a double-strain combination of Lactobacillus and Bifidobacterium in the studies reviewed. | - The study focuses on patients with systemic sclerosis (SSc), an autoimmune connective tissue disease characterized by fibrosis of the skin and internal organs, | - The systematic review included a total of 16 trials, with a control group size that varied across studies, as the sample sizes of primary studies ranged from 36 to 433 participants. |  | - The primary outcome of the systematic review and meta-analysis was to evaluate the effectiveness of probiotics in reducing anxiety symptoms in pregnant and lactating women, with a standardized mean difference (SMD) of 0.01 for pregnant women and -0.17 for lactating women, indicating a reduction in anxiety levels. | - Probiotics supplementation improved the gut microbiota of infants, indicating a positive impact on the overall health and development of newborns. | - The systematic review and meta-analysis utilized the Cochrane Collaboration guidelines for assessing the quality of included trials, employing the risk of bias tool (ROB-2) to evaluate the methodological quality of the studies analyzed. - Data from the primary studies were quantitatively synthesized, with standardized mean differences (SMD) calculated for anxiety and depression outcomes in pregnant and lactating women, along with confidence intervals (CI) and I² statistics to assess heterogeneity among the studies. | - The systematic review and meta-analysis did not report any specific adverse events associated with the administration of probiotics to pregnant and lactating women or their infants. - The focus of the study was on the effectiveness of probiotics in reducing anxiety and depression symptoms, as well as improving gut microbiota and alleviating gastrointestinal issues in newborns, without mentioning any negative side effects. | - The systematic review and meta-analysis included a total of 16 trials with varying sample sizes, but the specific follow-up duration for each trial was not detailed in the provided information. - The study focused on the effectiveness of probiotics on anxiety and depression symptoms in pregnant and lactating women, as well as the microbiota of newborns, without specifying the duration of follow-up assessments for the participants. | - The systematic review and meta-analysis reported standardized mean differences (SMD) for the effects of probiotics on anxiety and depression symptoms in pregnant and lactating women. For anxiety, the SMD for pregnant women was 0.01 and for lactating women was -0.17, indicating a minimal effect on anxiety levels. - For depression, the SMD for pregnant women was 0.05 and for lactating women was -0.10, suggesting that probiotics had a negligible impact on reducing depression symptoms in both groups. The confidence intervals for these effects indicate a lack of significant improvement in mental health outcomes. | - The p-value for the reduction of anxiety in pregnant women was reported as P = 0.04, indicating a statistically significant effect of probiotics on anxiety levels in this group. - For lactating women, the p-value for anxiety reduction was P = 0.98, suggesting no significant effect of probiotics on anxiety levels in this population. | - The heterogeneity measure (I²) indicates the percentage of variation across studies that is due to heterogeneity rather than chance. In this review, the I² values for anxiety in pregnant women was 70%, suggesting substantial heterogeneity, while the I² for lactating women was 0%, indicating no heterogeneity in that group. - For depression, the I² value for pregnant women was 40%, indicating moderate heterogeneity, while for lactating women, the I² was 60%, suggesting substantial heterogeneity in the results for that group as well. |
| 16 | M. Sakamoto, at al,2023 | The study involved two groups of patients: 29 patients with established systemic sclerosis (SSc) and 21 patients classified as very early systemic sclerosis (VEDOSS). The SSc group consisted of 27 females with a mean disease duration of 13.1 years, while the VEDOSS group included 19 females with a mean disease duration of 6.4 years. | - The study involved a total of 50 patients, with 29 patients diagnosed with systemic sclerosis (SSc) and 21 patients classified as very early systemic sclerosis (VEDOSS). | - The mean disease duration for the established systemic sclerosis (SSc) patients was 13.1 years, which translates to approximately 4,800 days (13.1 years x 365 days/year). | The study included a total of 50 patients, comprising 29 patients with established systemic sclerosis (SSc) and 21 patients classified as very early systemic sclerosis (VEDOSS). | - The study observed an increased abundance of the genus Lactobacillus in systemic sclerosis (SSc) patients, | - The study focuses on patients with systemic sclerosis (SSc), an autoimmune connective tissue disease characterized by fibrosis of the skin and internal organs, | - The control group consisted of healthy controls (HC) with 4 participants, aged 40 to 53 years, |  | - The primary outcome of the study was to investigate the differences in the taxa composition of the fecal microbiota between very early systemic sclerosis (VEDOSS) patients and established systemic sclerosis (SSc) patients, revealing distinct microbiota profiles in both groups. | - The study investigated gastrointestinal (GI) involvement and quality of life in patients with systemic sclerosis (SSc) and very early systemic sclerosis (VEDOSS) using the UCLA-Gastrointestinal Tract Questionnaire (GIT) 2.0 and the Short Form Health Survey-36 (SF36). It was found that VEDOSS patients had significantly higher SF-36 physical component scores compared to SSc patients, indicating better physical health status. | - The study utilized 16S rRNA Next Generation Gene-Sequencing analysis to assess the microbiota composition in fecal samples from patients with systemic sclerosis (SSc) and very early systemic sclerosis (VEDOSS). This method allows for detailed profiling of microbial taxa present in the samples. - Statistical analyses included differential abundance analysis using the DESeq2 function, which provided shrinked Log2 fold change values to compare the microbiota profiles between SSc and VEDOSS patients. Additionally, t-tests were employed to analyze differences in biomarker concentrations between SSc patients and healthy controls after stimulation with interleukin-17 (IL-17) and tumor growth factor-beta (TGF-b). | - The study did not report any adverse events related to the microbiota assessment or the gastrointestinal involvement in systemic sclerosis (SSc) and very early SSc (VEDOSS) patients. - There was a mention of poor results from lactobacillus-based probiotic treatment in literature, suggesting that the increased Lactobacillus values in SSc patients may indicate more severe gastrointestinal tract involvement, but specific adverse events were not detailed in the findings. | - The mean disease duration for the established systemic sclerosis (SSc) patients was 13.1 years with a standard deviation of 9.2 years. - The mean disease duration for the very early systemic sclerosis (VEDOSS) patients was 6.4 years with a standard deviation of 5.2 years. |  | - The study found statistically significant differences in the fecal microbiota composition between systemic sclerosis (SSc) patients and very early systemic sclerosis (VEDOSS) patients, with all results having a p-value < 0.05, indicating strong evidence against the null hypothesis. - In the comparison of quality of life scores, VEDOSS patients had a significantly higher SF-36-physical component summary score than SSc patients, with a p-value of <0.01, while the SF-36-mental component summary score showed a trend towards significance with a p-value of 0.06. |  |
| 17 | Sofie, Ingdam, Halkjær., at al,2023 | The population type in the study consisted of patients diagnosed with irritable bowel syndrome (IBS), which is the most prevalent gastrointestinal disorder in developed countries. | - The systematic review included eight randomized controlled trials (RCTs) with a total of 484 participants, |  | - The systematic review included a total of eight randomized controlled trials (RCTs) with 484 participants. |  | - The patients in the study are suffering from Irritable Bowel Syndrome (IBS), | - The control group in the study consisted of 484 participants, with 45 participants reporting adverse events.  - The serious adverse events in the control group included two occurrences, resulting in a risk ratio of 0.42 (95%CI: 0.07-2.60) when compared to the FMT group. |  | - The primary outcome of the study was the number of patients with improvements in symptoms, which were measured using a validated, global IBS symptoms score. | - The secondary outcomes assessed in the study included changes in quality-of-life scores for patients with irritable bowel syndrome (IBS) following fecal microbiota transplantation (FMT) compared to placebo. | - Risk ratios (RR) and corresponding 95% confidence intervals (CI) were calculated for dichotomous outcomes, while mean differences (MD) and 95% CI were calculated for continuous outcomes to assess the effectiveness of FMT compared to placebo in treating IBS symptoms. - The Cochrane risk of bias tool was utilized to evaluate the quality of the trials, and GRADE criteria were applied to assess the overall quality of the evidence regarding the efficacy and safety of FMT for IBS. | - Adverse events were reported in 97 participants in the FMT group and in 45 participants in the placebo group, indicating a higher incidence of adverse events in the FMT group (RR 1.17, 95%CI: 0.63-2.15). - One serious adverse event occurred in the FMT group, while two serious adverse events were reported in the placebo group, suggesting a lower risk of serious adverse events associated with FMT compared to placebo (RR 0.42, 95%CI: 0.07-2.60). | - The follow-up duration for assessing the efficacy of fecal microbiota transplantation (FMT) in treating irritable bowel syndrome (IBS) was three months after treatment. - The review included eight randomized controlled trials (RCTs) with a total of 484 participants, all of which evaluated the improvements in IBS symptoms at this three-month follow-up period. | - The systematic review and meta-analysis did not provide specific calculations for effect size, such as Cohen's d, in relation to the efficacy of fecal microbiota transplantation (FMT) for treating irritable bowel syndrome (IBS). Instead, it reported risk ratios (RR) and mean differences (MD) for various outcomes. - The results indicated that FMT did not show a significant benefit in improving IBS symptoms compared to placebo, with a risk ratio of 1.19 and a mean difference of -6.30 for quality of life, suggesting that the overall evidence regarding the effectiveness of FMT for IBS is insufficient and of low quality. | - The systematic review and meta-analysis did not provide specific p-values for the outcomes measured, such as the improvement in IBS symptoms or quality of life scores. Instead, it reported risk ratios (RR) and mean differences (MD) along with their corresponding 95% confidence intervals (CI) to assess the effectiveness of fecal microbiota transplantation (FMT) compared to placebo. - The overall quality of the evidence was deemed low due to factors such as moderate-high inconsistency, a small number of participants in the studies, and imprecision, which suggests that the findings should be interpreted with caution, but no specific p-values were mentioned to quantify the statistical significance of the results. | - The overall quality of the evidence regarding the efficacy and safety of fecal microbiota transplantation (FMT) for irritable bowel syndrome (IBS) was assessed as low due to moderate-high inconsistency among the studies included in the review. - The presence of moderate-high inconsistency suggests that the results of the trials varied significantly, which may impact the reliability of the conclusions drawn about FMT's effectiveness for IBS. |
| 18 | Mancai Wang,  at al,2023 | The population type included in the systematic review and meta-analysis consisted of patients diagnosed with moderate to severe irritable bowel syndrome (IBS), specifically focusing on IBS-D (diarrhea-predominant IBS) and IBS-M (mixed-diarrhea-and constipation IBS), which accounted for approximately 81.7% of the patients in the studies analyzed. | - The systematic review and meta-analysis included nine randomized controlled trials (RCTs) that assessed the efficacy and safety of fecal microbiota transplantation (FMT) for patients with irritable bowel syndrome | - The duration of capsule FMT in the included RCTs varied, with one study administering 25 capsules per day for 12 days, another for 3 days, one for 1 day with 19 capsules, and another for 3 days with 30 capsules. | This sample size was noted to be significantly smaller than the optimal information size |  | - The patients in the study were diagnosed with irritable bowel syndrome (IBS), | - The systematic review included nine randomized controlled trials (RCTs) that compared fecal microbiota transplantation (FMT) with placebo in patients with irritable bowel syndrome (IBS). The control group consisted of participants receiving placebo treatments, which were either autologous FMT or placebo capsules. |  | - The primary outcome of the study was the IBS-SSS (Irritable Bowel Syndrome Severity Scoring System) score, which was assessed at different time points after fecal microbiota transplantation (FMT) to evaluate the effectiveness of the treatment in improving IBS symptoms. | - The secondary outcomes of the study included the clinical response rate, which was defined by the relief level of IBS symptoms assessed using the IBS severity scoring system (IBS-SSS), Gastrointestinal Symptom Rating Scale for IBS (GSRS-IBS), or a daily symptom diary. This outcome aimed to evaluate how effectively FMT alleviated symptoms in patients with IBS over time. | - The authors conducted a meta-analysis using RevMan software and performed sensitivity analysis and meta-regression with Stata software. They utilized random-effects models to calculate mean difference (MD) or standardized mean difference (SMD) with 95% confidence intervals (CI) for continuous data, and risk ratios (RR) with 95% CI for dichotomous data. - The quality of evidence was assessed using the GRADE system, and the risk of bias for each included study was evaluated with the Cochrane Collaboration's tool as outlined in the Cochrane Handbook for Systematic Reviews of Interventions. | - The majority of adverse events reported in the randomized controlled trials (RCTs) were mild and self-limiting gastrointestinal symptoms, including abdominal pain, nausea, diarrhea, constipation, bloating or flatulence, headache, fatigue, and fever. Only one serious adverse event was noted, where a participant experienced transient vertigo and nausea during colonoscopy and was admitted for a few hours of observation. - Meta-analysis indicated that there were no significant statistical differences in the incidence of adverse events between the fecal microbiota transplantation (FMT) group and the placebo group. However, after sensitivity analysis, it was suggested that FMT may increase the incidence of abdominal pain, constipation, and diarrhea. | - The follow-up duration in the included randomized controlled trials (RCTs) varied between 10 weeks and 52 weeks, indicating a range of time over which the effects of fecal microbiota transplantation (FMT) were assessed in patients with irritable bowel syndrome (IBS). - Specific follow-up time points reported in the studies included assessments at 1 month, 3 months, 6 months, 24 months, and 36 months, allowing for a comprehensive evaluation of the long-term efficacy and safety of stool FMT compared to placebo. | - The study reported mean differences (MD) for continuous data, such as the IBS-SSS score, which were calculated using random-effects models. For example, a significant decrease in the IBS-SSS score was observed at various follow-up periods (1 month, 3 months, 6 months, 24 months, and 36 months) when comparing stool FMT to placebo, indicating a positive effect of stool FMT on IBS symptoms. - The risk ratios (RR) for dichotomous data were also provided, showing an increase in the clinical response rate at 3 months, 24 months, and 36 months post-FMT. However, the study did not provide specific calculations for Cohen's d, which is a standardized measure of effect size, indicating that the focus was primarily on mean differences and risk ratios rather than Cohen's d. | - The p-value is used to determine the statistical significance of the differences observed in the studies included in the meta-analysis. For instance, a p-value of less than 0.05 indicates that the differences between the FMT and placebo groups are statistically significant, while a p-value greater than 0.05 suggests no significant difference. - In the context of the studies analyzed, significant heterogeneity was identified among the studies, with a p-value threshold of 0.1 used to assess this heterogeneity. This means that when the p-value was below this threshold, it indicated significant variability in the results across different studies, which could affect the overall conclusions drawn from the meta-analysis. | - Heterogeneity among the studies was assessed using the I² statistic, where an I² value of 50% or higher indicated significant heterogeneity. In the meta-analysis, significant heterogeneity was observed, particularly in the clinical response rate and IBS-SSS score outcomes, prompting sensitivity analyses to identify and remove studies contributing to this heterogeneity. - After conducting sensitivity analyses, the results remained consistent, indicating that even after removing studies with obvious heterogeneity, the main outcomes for stool FMT compared to placebo still showed statistically significant differences, while the capsule FMT did not demonstrate significant differences from the placebo group at various time points. |
| 19 | Jie Gao,  at al,2023 | The population affected by depression includes nearly 350 million people worldwide, with a prevalence rate of approximately 4.4% in both developed and developing countries. In China, more than 95 million people suffer from depressive disorders, accounting for 6.8% of the total population. |  | - The duration of probiotic treatment for depression and its comorbidities is often specified in clinical studies, with some randomized controlled trials (RCTs) indicating treatment periods ranging from 8 weeks to | - The text does not provide specific information regarding the sample size used in the studies | - **Lactobacillus spp.**: This genus includes several strains such as L. plantarum, L. fermentum, L. rhamnosus, and L. casei, which are widely used as probiotics. | - Patients with inflammatory bowel disease (IBD), which includes Crohn's disease (CD) and ulcerative colitis (UC), |  |  | - The primary outcome of the studies reviewed is the alleviation of depressive symptoms in participants, which has been demonstrated through various randomized controlled trials (RCTs) showing that probiotics can effectively reduce depressive symptoms in individuals both with and without a clinical diagnosis of depression. | - The secondary outcomes of probiotic interventions in depression treatment may include the modulation of inflammatory markers, such as the reduction of pro-inflammatory cytokines and the enhancement of anti-inflammatory responses, which can contribute to alleviating depressive symptoms and improving overall mental health. |  | - Among the probiotic strains studied, L. plantarum ranked first in improving the quality of life of patients with IBS and had the lowest incidence of adverse events, indicating its safety profile in clinical use. - While probiotics like B. coagulans MTCC 5856 showed robust efficacy in treating IBS symptoms and major depressive disorder, the overall evidence for the efficacy of probiotic interventions in patients with IBS and depression remains limited, suggesting that the benefits of probiotics tend to be symptom- and strain-specific, which may also imply varying rates of adverse events across different strains. | - There is an urgent need for more rigorous randomized controlled trials (RCTs) with extended follow-up durations to assess the long-term safety and efficacy of probiotic supplementation in patients diagnosed with depression, both with and without comorbidities. This includes evaluating relapse rates after treatment. - The follow-up duration should also consider the optimal probiotic supplement content and dosage, as well as the specific patient population and disease severity threshold for inclusion in the studies. | - The paper discusses the potential therapeutic effects of probiotics on depression and its comorbidities, suggesting that probiotics can exert antidepressant effects through various mechanisms, including the modulation of gut microbiota, production of beneficial metabolites, and regulation of the immune system. However, specific effect sizes such as Cohen's d are not provided in the text. - The review highlights that while several randomized controlled trials have demonstrated the efficacy of probiotics in alleviating depressive symptoms, the benefits are strain-specific and may vary in magnitude. Detailed statistical measures like effect sizes for individual studies or specific probiotic strains are not mentioned in the provided information. |  |  |
| 20 | Supriya, Roy., at al,2023 | The population type discussed in the context of the paper primarily includes individuals suffering from inflammatory bowel disease (IBD), specifically those with active Crohn’s disease and ulcerative colitis/pouchitis. |  |  |  | - Probiotic species such as Lactobacillus and Bifidobacterium have been reported to exhibit several mechanisms of action, | - The paper discusses the management of inflammatory bowel disease (IBD), |  |  | - The primary outcome of using prebiotics, probiotics, and synbiotics in the management of inflammatory bowel disease (IBD) is the suppression of inflammation, induction of remission, and prevention of relapse in conditions such as Crohn’s disease and ulcerative colitis. This therapeutic approach aims to restore the balance between the gut microflora and the host's defense mechanisms in the intestinal mucosa. |  |  | - The text indicates that the therapeutic approach using prebiotics, probiotics, and synbiotics for managing inflammatory bowel disease (IBD) has been associated with minimal side effects, suggesting a favorable safety profile for these treatments. - However, it also notes that clinical trials have produced some conflicting results regarding the efficacy of these treatments, which implies that while adverse events may not be prominently reported, the variability in clinical outcomes necessitates further investigation into their effects on IBD remission and prevention. |  |  |  |  |
| 21 | Qin Zhang,  at al,2023 | The population type in the studies included in the meta-analysis consisted of patients with a clinical diagnosis of depression, specifically those diagnosed based on DSM-IVV, ICD-10, or validated depression rating scales. A total of 786 participants were involved, with most being female, as the percentage of females was reported to be over 50% in all studies. | - The meta-analysis included a total of 13 randomized controlled trials (RCTs) with a combined sample size of 786 participants, focusing on the effects of prebiotics, probiotics, and synbiotics on depression. | - The intervention duration for the studies included in the meta-analysis ranged from 3 to 24 weeks, with specific subgroups analyzed based on treatment duration of fewer than 8 weeks showing significant beneficial effects on depressive symptoms.  - Studies were stratified into three subgroups based on treatment duration: 4 weeks, 4 to 8 weeks, and more than 8 weeks, with significant improvements observed in the 4-week (SMD -0.37) and 4 to 8 weeks (SMD -0.32) groups, while those receiving more than 8 weeks of therapy did not show significant improvement (SMD -0.28). |  | - Common probiotic species mentioned in the studies include **Lactobacillus casei**, **Lactobacillus acidophilus**, and various strains of **Bifidobacterium** such as **B. longum**, **B. bifidum**, and **B. breve**. These species are recognized for their beneficial effects on gut health and potential antidepressant-like effects.  - **Lactobacillus casei** is typically used to treat gastrointestinal diseases and has shown promise in improving depression-like behaviors in animal studies by reversing changes in brain-derived neurotrophic factor (BDNF) expression induced by chronic stress. **Bifidobacterium breve** has been noted for its antidepressant | - The patients included in the study had a clinical diagnosis of depression based on established criteria such as DSM-IVV, ICD-10 | The control group received an undistinguished placebo, allowing for a comparison of the effects of prebiotics, probiotics, and synbiotics on depression symptoms.  - In the studies analyzed, the control groups were specifically designed to assess the efficacy of the interventions by measuring changes in depressive symptoms using various rating scales before and after the intervention, ensuring that the results could be attributed to the treatments rather than other factors. |  | - The primary outcome of the included studies in the meta-analysis was the effectiveness of prebiotics, probiotics, and synbiotics in alleviating depressive symptoms, which was assessed through various scales measuring depression severity, such as the Hamilton Depression Rating Scale (HDRS) and the Beck Depression Inventory (BDI). | - The secondary objectives of the study included exploring influential factors associated with the effectiveness of prebiotics, probiotics, and synbiotics in alleviating depressive symptoms, as well as summarizing alterations in gut microbiota depicted by different indices and changes in depression-related biochemical indicators. | - The risk of bias in the included studies was evaluated using Cochrane Collaboration's Risk of Bias Tool 2, which assessed five domains: the randomization process, deviations from intended interventions, missing outcome data, measurement of the outcome, and selection of the reported result. This evaluation helped determine the overall bias in the studies. - A meta-analysis was performed using Stata SE 15.1 to evaluate the changes in inflammatory markers (IL-1b, IL-6, and TNF-a) after the intervention, and multiple meta-regression was conducted to interpret heterogeneity and evaluate the effects of different factors on the outcomes. Additionally, a sensitivity analysis was carried out to examine the robustness of the results, ensuring that no single study significantly impacted the overall findings. | - The meta-analysis did not report any significant differences in inflammatory markers such as IL-1b, IL-6, and TNF-a between the intervention groups (prebiotics, probiotics, synbiotics) and the placebo group, indicating that these treatments did not lead to notable adverse events related to inflammation. - The study did not provide specific information on adverse events experienced by participants during the interventions, suggesting that the focus was primarily on the efficacy of the treatments rather than documenting potential negative effects. | - The intervention duration for the studies included in the meta-analysis ranged from 3 to 24 weeks, with specific subgroups analyzed based on treatment duration of fewer than 8 weeks showing significant beneficial effects on depressive symptoms. - The assessment of depressive symptoms was evaluated both at the end of the intervention and after a follow-up period, indicating that the timing of the assessment could influence the outcomes, with significant improvements noted after both intervention and follow-up periods. | - The overall effect size for the improvement of depressive symptoms in patients receiving prebiotics, probiotics, or synbiotics compared to the placebo group was significant, with a standardized mean difference (SMD) of -0.34, indicating a moderate effect size in alleviating depression symptoms. - Subgroup analyses revealed that probiotics specifically had a significant effect size of -0.35, while prebiotics did not show a significant difference from the placebo group (SMD -0.25), suggesting that probiotics are more effective in reducing depressive symptoms compared to prebiotics. | - The p-values reported in the meta-analysis indicate the statistical significance of the effects of prebiotics, probiotics, and synbiotics on depressive symptoms. For instance, the overall pooled effect showed a significant improvement in depressive symptoms with a p-value of 0.001, suggesting strong evidence against the null hypothesis that these interventions have no effect compared to placebo. - Subgroup analyses also reported significant p-values for different conditions; for mild depression, the p-value was 0.002, and for moderate depression, it was 0.001, confirming that both groups benefited from the interventions. Additionally, the p-values for the efficacy of probiotics specifically were significant, with a pooled effect showing a p-value of 0.001, indicating that probiotics were particularly effective in alleviating depressive symptoms. | - Heterogeneity was assessed using the I² statistic, which measures the percentage of variation across studies that is due to heterogeneity rather than chance. In the meta-analysis, low heterogeneity was observed in several subgroups, such as for the Chao-1 index (I² = 0.0), Shannon index (I² = 8.3), and observed species (I² = 0.0), indicating consistent results across studies for these measures. - In the subgroup analysis based on the severity of depression, the I² values were also low, with mild depression showing I² = 20.8 and moderate depression showing I² = 21.8, suggesting that the treatment effects for both mild and moderate depression were consistent across the included studies. However, some subgroups, such as those with multiple strains of probiotics, exhibited higher heterogeneity (I² = 53.6), indicating variability in the treatment effects within that subgroup. |
| 22 | Ricardo Morales-Torr,  at al,2023 | The study involved a randomized placebo-controlled clinical trial with a sample of 135 healthy adults, predominantly women, recruited through social media in Santiago, Chile. This population was characterized as otherwise healthy, but the sample was biased towards a Mediterranean-like diet and lifestyle. | - The study utilized a randomized placebo-controlled clinical trial design, which included a sample size of 135 healthy adults. | - The randomized placebo-controlled clinical trial lasted for a total of 84 days, which included a 4-week (28 days) probiotic supplementation period followed by a follow-up measurement period. | The sample size for the study was calculated using the statistical program GPower, with parameters defined based on previous studies evaluating the effect of probiotics on well-being scales. | - The probiotic species tested in the study were Lactobacillus helveticus R0052 and Bifidobacterium longum R0175, | - The study focused on healthy adults and did not specifically address any particular diseases; instead, it examined the effects of probiotic supplementation on mental health outcomes such as anxiety and emotional | - The control group consisted of 72 participants who were randomly allocated to the placebo group. |  | - The primary outcome measured in the study was the Ryff Scales of Psychological Well-Being (RYFF), which showed a significant change over time, indicating that participants experienced an increase in well-being scores from baseline to follow-up, regardless of whether they received probiotic or placebo treatments. | - The secondary outcomes included five scales for measuring aspects related to well-being, which were used to evaluate the participants' overall psychological state. These scales comprised the Satisfaction with Life Scale (SWLS), the positive and negative subscales of the Positive and Negative Affect Schedule (PANAS), the Difficulties in Emotion Regulation Scale (DERS), and the mental and physical components of the Short Form Health Survey (SF-36). | - The study utilized a repeated measure ANOVA to analyze the effects of probiotic supplementation on well-being scales, allowing for the detection of significant differences across multiple time points (baseline, post, and follow-up) within the same subjects. - A linear mixed-effects model was employed to assess the interaction between treatment groups (probiotic vs. placebo), lifestyle habits, and time, controlling for variables such as age and gender, and including both fixed and random effects to account for individual differences among participants. | - In the probiotic group, three participants did not receive the treatment due to adverse events, which included starting an antibiotic treatment, starting another probiotic treatment, and being diagnosed with COVID-19. - In the placebo control group, one participant discontinued the treatment due to adverse effects, indicating that there were some reported adverse events during the trial. | - The follow-up duration in the study was 12 weeks after the baseline measurement, which was conducted at the beginning of the experiment. This means participants were assessed again after the initial 4-week probiotic or placebo intervention period. - Participants completed assessments at three different time points: baseline (before any intervention), post-intervention (4 weeks after baseline), and follow-up (12 weeks after baseline), allowing for the evaluation of changes over time in relation to the interventions. | - The sample size was calculated to detect an effect size of 0.88 using a statistical program, indicating a strong effect size based on the parameters defined for evaluating the effect of probiotics on well-being scales. This suggests that the study was adequately powered to identify significant effects if they existed. - Despite the strong effect size calculated for the sample, the results showed no significant effects of probiotic intake on the overall outcomes for the whole sample, highlighting a discrepancy between the expected effect size and the actual findings in the context of the study. | - A p-value of 0.05 was considered significant in the study, indicating that results with a p-value below this threshold would be deemed statistically significant. - For the correlation analyses, a more stringent criterion was applied, where a correlation was considered significant if its p-value was below or equal to 0.002, which corresponds to a Bonferroni correction for multiple comparisons. |  |
| 23 | Tatsuya, Dokoshi., at al,2023 | The study focuses on the impact of skin inflammation on the gut microbiome and intestinal antimicrobial activity, suggesting a connection between skin health and gut health. |  | - The randomized placebo-controlled clinical trial lasted for a total of 84 days, which included a 4-week (28 days) probiotic supplementation period followed by a follow-up measurement period. |  |  | - The study investigates how skin inflammation affects intestinal antimicrobial activity, |  |  | - The primary outcome of the study is the assessment of how skin inflammation affects intestinal antimicrobial activity, which is crucial for maintaining gut health and preventing infections. |  |  |  |  |  |  |  |
| 24 | Monika Jach, at al,2023 | The population type discussed in the context includes adults suffering from major depressive disorder (MDD) and metabolic depression, particularly highlighting the increased prevalence of depressive disorders in individuals with chronic physical conditions such as type 2 diabetes (T2D) and metabolic syndrome (MetS). | - The paper discusses various studies on the effects of probiotics on depression, but it does not provide specific details regarding the study design, | - For the effectiveness of antidepressant therapy, psychobiotics should be administered at a dose higher than 1 billion CFU/day for at least 8 weeks. | The research does not specify a sample size for the studies discussed regarding the role of probiotics and their metabolites in the treatment of depression. The focus is primarily on the relationship between intestinal microbiota and mental health rather than on specific experimental data or sample sizes.  - Additionally, the paper emphasizes the general findings from various studies without detailing the number of participants involved in those studies, making it difficult to ascertain a specific sample size related to the effects of probiotics on depression. | - The most commonly studied probiotic species with psychobiotic properties belong to the genera Bifidobacterium and Lactobacillus. | - Patients with depression often experience comorbid metabolic conditions, such as metabolic syndrome (MetS), type 2 diabetes mellitus (T2D), and non-alcoholic fatty liver disease (NAFLD), |  |  | - The primary outcome of the studies reviewed indicates that probiotic administration did not reveal a significant positive effect on mood in both healthy and depressive populations, although a slight reduction in depression scale scores was observed in cases of mild to moderate depression. | - The secondary outcome of probiotic administration in the context of depression treatment includes the reduction of depression scale scores, particularly noted in mild and moderate depression, indicating a potential benefit in alleviating depressive symptoms even if the overall mood improvement is not statistically significant across all studies. |  | - The text does not specifically mention any adverse events related to the administration of probiotics or psychobiotics in the treatment of depression. It primarily focuses on the positive effects and potential benefits of probiotics in alleviating depressive symptoms and improving gut-brain axis functions. - While the text discusses the relationship between gut microbiota and mental health, it does not provide information on any negative side effects or adverse reactions experienced by participants in the studies referenced. The emphasis is on the therapeutic potential of probiotics rather than any adverse outcomes. | - The administration of probiotics labeled as psychobiotics should be conducted at a dose higher than 1 billion CFU/day for a minimum duration of 8 weeks to observe improvements in mental disorders. - The follow-up duration for assessing the effectiveness of probiotics in alleviating depressive symptoms is not explicitly mentioned in the provided information, but the 8-week treatment period suggests that follow-up assessments could be conducted shortly after this duration to evaluate outcomes. |  |  |  |
| 25 | Emanuele Rinninella, at al,2023 | The population type studied in the review consisted of patients diagnosed with major depressive disorder (MDD), which is a common psychiatric disorder affecting millions worldwide and is associated with significant disability and health-related quality of life impairments. | - The systematic review included seven clinical trials involving patients with major depressive disorder (MDD). | - Most studies reviewed had a duration of four to eight weeks for probiotic intervention, which translates to approximately 28 to 56 days. | The systematic review identified seven clinical trials involving patients with major depressive disorder (MDD), but the small number of studies and heterogeneous sources of data limited the ability to perform a meta-analysis.  - The studies reviewed generally had small sample sizes, which contributed to the overall low-to-moderate risk of bias and made it difficult to draw firm conclusions regarding the effects of probiotic supplementation on gut microbiota and depressive symptoms. | - The review specifically mentions two probiotic genera, Lactobacillus and Bifidobacterium, | - The patients discussed in the study are individuals diagnosed with Major Depressive Disorder (MDD), | he specific group sizes for the control groups in these trials were not detailed in the provided information. The studies generally had a low-to-moderate risk of bias, primarily due to a lack of control for dietary effects on gut microbiota. |  | - The primary objective of the study was to investigate the effect of probiotic supplementation on gut microbiota in patients with major depressive disorder (MDD). This involved assessing changes in gut microbiota composition and diversity following probiotic intervention. | - The secondary outcome of the review focused on the change in depressive symptoms, which was assessed using validated rating scales such as the Hamilton Depression Rating Scale (HAM-D) or Beck Depression Index (BDI) after the probiotic intervention. The meta-analysis of five studies indicated a pooled standardized mean difference (SMD) of -0.50, suggesting that probiotic supplementation did not significantly improve depressive symptoms compared to placebo. | - The studies utilized a random effects meta-analysis to pool results where possible, calculating standardized mean differences (SMD) to express the size of the treatment effect in standard deviation units, accommodating the use of different scales and units across the trials reviewed. - The risk of bias in the studies was appraised using Version 2 of the Cochrane risk-of-bias tool for randomized trials (RoB 2), which evaluates several key domains such as sequence generation, allocation concealment, blinding, incomplete outcome data, selective outcome reporting, and other potential sources of bias inherent to the studies. | - There is a lack of systematic reporting on adverse events associated with probiotic supplementation in patients with major depressive disorder (MDD), making it difficult to assess the safety profile of these interventions comprehensively. - Although the available studies do not indicate an increased risk of serious side effects, anecdotal reports suggest that probiotics may worsen outcomes in some cases, and gastrointestinal disturbances such as diarrhea, abdominal pain, and bloating have been reported in fecal microbiota transplant recipients. | - The follow-up duration for probiotic intervention in the studies reviewed varied, with most studies implementing a duration of four to eight weeks. This short duration may not be sufficient to observe significant alterations in gut microbiota composition or clinical improvement in depressive symptoms. - The review suggests that patients with major depressive disorder (MDD) may require a longer time than eight weeks to show clinical improvement, indicating that the microbial host environment may also need more time to produce significant microbiota alterations. | - The systematic review calculated standardized mean differences (SMD) to express the size of the treatment effect in standard deviation (SD) units, as the studies used different scales and outcome measurements had varying units. This approach is similar to Cohen's d, which is commonly used to measure effect size in psychological research. - The review noted that previous meta-analyses indicated a small to moderate effect size for probiotic supplementation on depressive symptoms, emphasizing the need for further research to determine the optimal dose, duration, and specific strains of probiotics to achieve more significant effects. | - The systematic review found that probiotic supplementation did not significantly improve depressive symptoms compared to placebo, with a pooled standardized mean difference (SMD) for change in depressive rating scales reported as -0.50 (95% CI: -1.13 to 0.14). This indicates that the effect of probiotics on depressive symptoms was modest and not statistically significant. - The review highlighted that the small number of studies and heterogeneous data precluded a meta-analysis, and most trials had a low-to-moderate risk of bias, primarily due to a lack of control for dietary effects on gut microbiota. This suggests that the findings regarding the efficacy of probiotics in treating major depressive disorder may not be robust. | - The heterogeneity of the studies reviewed was indicated by an I² value of 69, suggesting a moderate level of inconsistency among the results of the included trials. This variability may stem from differences in study design, sample sizes, and the specific probiotic interventions used across the trials. - The presence of heterogeneity complicates the interpretation of the findings, as it reflects the diverse nature of the diagnosis of major depressive disorder (MDD) and the various factors influencing gut microbiota composition, such as diet, gender differences, and individual variations in microbiota. |
| 26 | Viktoriya Nikolova and Anthony Cleare. At al,2023 | The study involved adults diagnosed with major depressive disorder (MDD) who were also taking antidepressants, indicating a specific focus on individuals experiencing significant mental health challenges. | - The study design is a double-blind randomized placebo-controlled pilot trial that investigated the effects of an 8-week adjunctive multi-strain probiotic intervention in adults with major depressive disorder (MDD) who were taking antidepressants. The sample size included 49 participants, with 24 in the probiotic group and 25 in the placebo group.  - The publication year is not explicitly mentioned in the provided information, and the country of origin is also not specified. | - The duration of the probiotic intervention in the study was 8 weeks, which is equivalent to 56 days. | The study included a total of 49 participants who were randomized into two groups: 24 participants in the probiotic group and 25 participants in the placebo group. | - The study investigated an 8-week adjunctive multi-strain probiotic intervention, which included various probiotic species aimed at improving symptoms in adults with major depressive disorder (MDD) | - The study focuses on patients with Major Depressive Disorder (MDD) who are taking antidepressants, | - The control group in the study consisted of 25 participants who were randomized to receive a placebo treatment during the 8-week trial period. This group was compared to the probiotic group, which had 24 participants.  - The event for the control group involved the administration of a placebo, with assessments of psychiatric data, stool, and blood samples collected at baseline, week 4, and week 8 to evaluate the effects of the treatment compared to the probiotic intervention. |  | - The primary outcome of the study was the improvement in depressive and anxiety symptoms among participants with major depressive disorder (MDD) who received an 8-week adjunctive multi-strain probiotic intervention compared to those receiving a placebo. This was measured using standardized effect sizes (SES) from linear mixed models, showing significant improvements in scores on the Hamilton Depression Rating Scale (HAMD) and Hamilton Anxiety Rating Scale (HAMA) at both week 4 and week 8. | - The study assessed the impact of the probiotic intervention on anxiety symptoms, with significant improvements observed in the Hamilton Anxiety Rating Scale (HAMA) scores at both week 4 (SES [95%CI] = 0.67 [0.00, 0.95]) and week 8 (SES [95%CI] = 0.79 [0.06, 1.05]), indicating that probiotics may effectively reduce anxiety levels in individuals with major depressive disorder (MDD). | - The study utilized linear mixed models to calculate standardized effect sizes (SES) for assessing the improvements in depressive and anxiety symptoms between the probiotic and placebo groups at different time points (week 4 and week 8). This statistical approach allowed for the analysis of repeated measures data while accounting for individual variability. - Intent-to-treat analyses were performed, which included all randomized participants regardless of adherence to the treatment protocol. This method helps to minimize bias and provides a more accurate estimate of the treatment effect in real-world settings. | - There were no serious adverse reactions reported during the trial, indicating a high level of safety for the probiotic intervention among participants with major depressive disorder (MDD). - The study recorded an attrition rate of 8%, with 3 participants from the placebo group and 1 from the probiotic group dropping out, suggesting that the treatment was well-tolerated by the majority of participants. | - The follow-up duration for the study investigating the effects of probiotics in adults with major depressive disorder (MDD) was 8 weeks, during which participants were assessed at baseline, week 4, and week 8. - Throughout the follow-up period, psychiatric data, as well as stool and blood samples, were collected to evaluate the efficacy and underlying mechanisms of the probiotic intervention. | - The study reported standardized effect sizes (SES) from linear mixed models, indicating that the probiotic group experienced greater improvements in depressive symptoms, with a Cohen’s d equivalent of 0.70 at week 4 and 0.64 at week 8, compared to the placebo group. This suggests a moderate to large effect of probiotics on reducing depressive symptoms in individuals with major depressive disorder (MDD). - For anxiety symptoms, the effect sizes were also significant, with Cohen’s d values of 0.67 at week 4 and 0.79 at week 8 for the probiotic group, indicating a moderate to large effect on anxiety reduction compared to the placebo group. These findings highlight the potential efficacy of probiotics as an adjunctive treatment in MDD. | - The study reported specific statistical significance for the increase in levels of Bacillaceae, with a false discovery rate (FDR) p-value of less than 0.05, indicating a statistically significant correlation between this specific taxon and reductions in anxiety scores among participants in the probiotic group. - The results also indicated that the probiotic group showed greater improvements in depressive and anxiety symptoms, with standardized effect sizes (SES) calculated from linear mixed models, but specific p-values for these outcomes were not provided in the abstract or full text. |  |
| 27 | Jennifer, C, Chan., at al,2023 | The study focuses on individuals experiencing acne, which is a common skin condition affecting various demographics, particularly adolescents and young adults. |  |  |  |  | - The case study focuses on acne, which is a common skin condition characterized by the presence of pimples |  |  |  |  |  |  |  |  |  |  |
| 28 | Michalina Sikorska,  at al,2023 | The review included three distinct population types: healthy participants with subclinical depressive or anxiety symptoms, depressed patients, and patients with depression and comorbid somatic illnesses. This categorization allowed for an analysis of the effectiveness of probiotic treatment across different levels of depressive symptoms and the presence of additional health conditions. | - The systematic review included a total of 20 clinical trials, with 17 being double-blind randomized placebo-controlled trials and 2 open-label randomized controlled trials. | - The duration of the studies included in the review ranged from 4 to 12 weeks, which translates to approximately 28 to 84 days. | - A total of 19 clinical trials were evaluated in the systematic review, which included a combined group of 1461 participants. The group sizes in these studies ranged from 11 to 156 participants each.  - The systematic review identified a total of 5819 citations, out of which 2714 did not meet the inclusion criteria and were excluded before screening. After removing duplicates, 2999 articles were left, and ultimately, 20 records met the inclusion criteria for the review. | - The probiotic species administered in the studies included various strains from the genera Bacillus, Bifidobacterium, Lacticaseibacillus, Lactobacillus, Lactococcus, and Streptococcus. | - The systematic review focuses on patients suffering from depression | - The studies included in the systematic review comprised a total of 1461 participants, with group sizes for the control (placebo) groups ranging from 11 to 156 participants across the various trials. |  | - The primary outcome of the systematic review was to evaluate the effectiveness of probiotic therapy in reducing depressive symptoms in patients with major depressive disorder and those with subclinical depressive or anxiety symptoms, focusing on the molecular mechanisms involved in this process. | - The review indicates that most studies evaluating the efficacy of probiotics in treating depression often considered the effect on immune parameters as secondary outcomes. This suggests that while the primary focus was on the impact of probiotics on depressive symptoms, researchers also measured changes in immune-related markers to assess the broader effects of probiotic therapy. | - The standardized mean difference (SMD) with 95% confidence intervals (CI) was calculated to assess the effects of probiotic administration on various parameters, including BDNF levels and inflammatory markers, in both depressed patients and healthy participants with subclinical symptoms of depression or anxiety. - The systematic review followed PRISMA guidelines, which included a comprehensive search strategy and the evaluation of clinical trials, ensuring that the data analysis was structured and adhered to established reporting standards for systematic reviews and meta-analyses. | - No serious adverse effects were reported in any of the studies described in the systematic review and meta-analysis of randomized clinical trials evaluating the effects of probiotics on depressive symptoms. - The safety of probiotic therapy was emphasized, indicating that the administration of probiotics did not lead to significant negative outcomes in the patient populations studied, including those with major depressive disorder and comorbid somatic conditions. | - The duration of the studies included in the review ranged from 4 to 12 weeks, indicating a variety of follow-up periods among the clinical trials assessing the effectiveness of probiotics in treating depression. - Future randomized clinical trials (RCTs) are suggested to have a medium long-term duration of at least 8 weeks to better evaluate the long-term effectiveness of probiotics in treating depression and preventing its recurrence. | - The standardized mean difference (SMD) was calculated in the systematic review, with a significant increase in Brain-Derived Neurotrophic Factor (BDNF) levels during probiotic treatment compared to placebo, resulting in an SMD of 0.37 (95% CI [0.07, 0.68], p = 0.02). This indicates a moderate effect size suggesting that probiotics may have a positive impact on BDNF levels in depressed patients. - For C-reactive protein (CRP) levels, the SMD was found to be -0.47 (95% CI [-0.75, -0.19], p = 0.001), indicating a significant reduction in CRP levels among probiotic-treated patients compared to the placebo group. This negative effect size suggests that probiotics may help in reducing inflammation associated with depression, particularly in patients with comorbid somatic illnesses. | - The p-value for the increase in BDNF levels during probiotic treatment compared to the placebo was found to be 0.02, indicating a statistically significant effect of probiotics on BDNF levels in depressed patients with or without comorbid somatic illnesses. - For the reduction in CRP levels in probiotic-treated patients compared to the placebo, the p-value was 0.001, demonstrating a significant decrease in inflammation markers among depressed patients with somatic comorbidities. | - Heterogeneity between studies was measured using Tau-square, chi-square, and I-square statistics, with an I-square value of 50 considered to indicate significant heterogeneity. This statistical approach helps to assess the variability in study outcomes and the extent to which the results of the studies differ from one another. - Subgroup analyses to assess whether results were influenced by the group (healthy subjects, patients with depression without comorbidities, and patients with depression with somatic comorbidities) were not feasible due to the lack of studies in the respective subgroups, which further complicates the interpretation of heterogeneity in the findings. |
| 29 | Sabbir Khan, at al,2023 | The studies referenced in the review primarily focus on both human populations and experimental rodent models to investigate the effects of short-chain fatty acids (SCFAs) on inflammatory skin diseases. Clinical studies have shown differences in SCFA levels between patients with chronic spontaneous urticaria (CSU) and healthy individuals, indicating a direct correlation with human health conditions. | - The review discusses various studies related to short-chain fatty acids (SCFAs) and their role in inflammatory skin diseases, particularly focusing on atopic dermatitis, urticaria, and psoriasis. However, specific details regarding the study design, publication year, country of origin, and sample size of each included study are not provided in the text.  - The text emphasizes the correlation between SCFAs and inflammatory skin diseases, mentioning findings from animal models and human studies, but it does not enumerate or detail individual studies with their respective design characteristics or demographic information. |  | - The review discusses various studies that indicate a correlation between short-chain fatty acids (SCFAs) and inflammatory skin diseases, particularly focusing on atopic dermatitis (AD), eczema, and psoriasis. However, it does not specify the sample sizes used in these studies, nor does it provide quantitative data regarding the number of participants involved in the research.  - The text mentions findings related to SCFA levels in infants and young children with eczema and AD, indicating lower levels of SCFAs in these populations compared to healthy controls. Yet, it emphasizes the need for more clinical evidence to elucidate the correlation between SCFAs and eczema, suggesting that the sample sizes in existing studies may not be sufficient to draw definitive conclusions. | - Probiotic Staphylococcus epidermidis can inhibit bacterial colonization and inflammation caused by Propionibacterium acnes (P. acnes) | - Patients with inflammatory skin diseases such as atopic dermatitis (AD), psoriasis, eczema, and chronic spontaneous urticaria (CSU) |  |  |  |  |  |  |  |  |  |  |
| 30 | Rosanna Di, at al,2022 | The population type studied includes individuals with cardiovascular diseases (CVD), such as those with hypertension, coronary heart disease, and stroke. Specific studies have focused on spontaneously hypertensive patients, heavy smokers, and individuals with Type II diabetes mellitus and pre-diabetes. | - The duration of probiotic supplementation in some studies mentioned is 12 weeks, which translates to approximately 84 days. | - The duration of probiotic supplementation in some studies mentioned is 12 weeks, which translates to approximately 84 days. This duration was used to observe the effects on biomarkers of inflammation and | - The article mentions that due to the variable quality and sample sizes of multiple clinical investigations, it is not possible to evaluate the efficacy and safety of probiotic medication combined with enteral nutrition (EN) in a systematic manner. This indicates that sample sizes in the studies reviewed may vary significantly, affecting the reliability of the findings.  - Specific sample sizes for individual studies are not provided in the text; however, it does reference a study involving 81 obese postmenopausal Caucasian women, which suggests that sample sizes can differ widely across different clinical trials examining the effects of probiotics and prebiotics on cardiovascular health. | - Probiotic species such as Lactobacillus plantarum and Lactobacillus rhamnosus GG have been shown to improve endothelial function and reduce systemic inflammation in individuals with cardiovascular disease, | - Cardiovascular disease (CVD) encompasses a range of disorders affecting the heart and blood vessels, including hypertension, coronary heart disease, heart failure, angina pectoris, myocardial infarction, and vascular strokes. It is associated with low-grade inflammation and is a leading cause of death globally, particularly in middle-aged and elderly populations.  - The gut microbiome plays a significant role in the development of cardiovascular disease, with imbalances linked to increased production of harmful metabolites like N-trimethylamine oxide, which can contribute to adverse cardiovascular outcomes. Probiotics and prebiotics may offer therapeutic benefits by improving gut microbiota composition and reducing inflammation, potentially mitigating the risk factors associated with CVD. |  |  | - The primary outcome of the study is to examine the beneficial effects of probiotic and prebiotic organisms on cardiovascular health, specifically focusing on conditions such as heart disease, coronary heart disease, stroke, and hypertension. | - Secondary outcomes in the context of probiotics and prebiotics for cardiovascular disease may include improvements in biomarkers of inflammation and metabolic profiles, such as reductions in high-sensitivity C-reactive protein, nitric oxide, and low-density lipoprotein cholesterol. These outcomes are significant as they can indicate a decrease in systemic inflammation and a potential reduction in cardiovascular risk. |  | - The text mentions that the application of enteral nutrition (EN) in patients who have suffered a major stroke can lead to complications such as diarrhea, constipation, and infection within one to two weeks of initiating therapy, which may reduce the effectiveness of EN. - Caution is advised regarding the human application of prebiotics, particularly inulin, due to observed deleterious effects on cardiovascular disease, indicating potential adverse events associated with their use. | - The follow-up duration for the studies mentioned in the text varies, with some clinical trials administering probiotics for a period of 12 weeks to observe changes in biomarkers related to cardiovascular disease and metabolic syndrome. - The text does not specify a uniform follow-up duration across all studies, indicating that the duration may differ based on the specific research design and objectives of each clinical investigation. |  |  |  |
| 31 | Yongqiong Deng,  at al,2023 | The study involved 46 patients diagnosed with moderate to severe acne vulgaris, aged between 18 to 30 years, who were treatment-naive and had not taken any systemic antibiotics, retinoids, corticosteroids, or immunosuppressive agents within six months prior to the study. | - The study was designed as a randomized controlled trial involving 46 patients diagnosed with moderate to severe acne vulgaris, conducted from June 2019 to April 2020 in Luzhou city, Sichuan province, China. | - The treatment duration for the acne patients in the study was three months, which is approximately 90 days. | - A total of 46 patients diagnosed with moderate to severe acne vulgaris were initially allowed into the study, but only 40 patients were analyzed after some participants withdrew or were removed due to various | - The study identified significant changes in the gut microbiota composition in patients with acne vulgaris after treatment with metformin, | - The study focused on patients diagnosed with moderate to severe acne vulgaris, a chronic inflammatory skin disorder that affects approximately 85% of adolescents and can lead to permanent physical scarring and a reduced quality of life.  - The severity of acne vulgaris in the patients was assessed using the Global Acne Grading System (GAGS), with a GAGS score of 19 defined as moderate to severe acne vulgaris, indicating the need for effective treatment options. | - The control group in the study consisted of seven-week-old male Sprague Dawley (SD) rats that were injected with normal saline, serving as the model control for comparison against the treatment groups receiving metformin and isotretinoin.  - Each experimental group, including the control group, had three rats, and every experiment was repeated three times to ensure the reliability of the results. |  | - The primary outcome of the study was the reduction in the severity of acne vulgaris, which was evaluated using the Global Acne Grading System (GAGS) scores. Both treatment groups (those receiving isotretinoin alone and those receiving isotretinoin with metformin) showed significant decreases in GAGS scores after treatment, indicating an improvement in acne severity. | - The secondary outcome of the study included the evaluation of the gut microbiota composition and diversity in patients with acne vulgaris, as assessed through 16S rDNA sequencing of fecal samples. The results indicated a significant decrease in microbial diversity in both the acne treatment groups (AVP and AVM) at baseline compared to healthy controls, with improvements in diversity observed in the AVM group after metformin treatment. | - The data were analyzed using SPSS version 21, with over 90 variables showing abnormal distribution as determined by the Shapiro-Wilk test. Consequently, the Kruskal-Wallis rank-sum and Wilcoxon rank-sum tests were employed for analysis, and results were reported as mean ± standard deviation (SD). - Statistical significance was defined as a p-value of less than 0.05, and the GraphPad Prism 8.0 software package was utilized for plotting the graphs to visually represent the data findings. | - The study highlights concerns regarding the adverse effects of isotretinoin, which include cheilitis, xerosis, acne flare, photophobia, elevated liver enzymes, and depressed mood. These side effects have raised the need for new treatment methods for acne vulgaris. - While the paper discusses the therapeutic effects of metformin as an adjunct therapy for acne vulgaris, it does not specifically report any adverse events associated with metformin treatment in the study participants. | - The follow-up duration for patients in the study was 12 weeks, during which they were monitored for improvements in acne severity and adherence to the treatment protocol. - Patients were followed up every month during the 12-week treatment period, and their pills were counted during each visit to confirm adherence to the study protocol. |  | - The statistical significance was defined as P < 0.05, indicating that any p-value below this threshold would be considered statistically significant in the study's analyses. - Various p-values were reported throughout the study, such as P < 0.001 for the increase in the relative abundances of Bacteroides and Bifidobacterium after metformin treatment, and P = 0.018 indicating that the GAGS scores for the AVM group were lower than those for the AVP group after treatment. |  |
| 32 | Oliwia Gawlik-Kotelnicka,  at al,2023 | The study population consisted of 60 adult patients diagnosed with depressive disorders, such as depressive episode, recurrent depression, and mixed depressive and anxiety disorder, recruited from psychiatric outpatient clinics and through social media advertisements in central Poland. | - The study design is a four-arm, parallel-group, prospective, randomized, double-blind, controlled trial, specifically designed as an internal feasibility and pilot study as part of a larger randomized controlled trial (RCT) on the effects of probiotic supplementation in patients with depressive disorders.    - The study was conducted in 2023 in central Poland, with a sample size of 60 participants recruited from psychiatric outpatient clinics and through social media advertisements. | - The duration of the intervention period for the study was 60 days, during which participants received either the probiotic preparation or a placebo. | - The study population consisted of 60 patients recruited from psychiatric outpatient clinics and through social media advertisements, which was designed to be large enough to provide sufficient information on the chosen feasibility outcome measures. | - The probiotic preparation used in the study contained two specific strains: Lactobacillus helveticus Rosell®-52 and Bifidobacterium longum Rosell®-175. | - The study focused on adult patients diagnosed with depressive disorders, | - The control group (PLC) consisted of 30 participants, as the total study population was 60, split evenly between the probiotic group (PRO) and the placebo group (PLC). |  | - The primary outcome measure of the study was the rate of eligibility per month, which was assessed as the number of subjects eligible for the study each month relative to those who were assessed for eligibility. An eligibility ratio was constructed to show the proportion of subjects meeting the eligibility criteria. | - The secondary outcome measures included rates of recruitment, enrolment per month, retention, and adherence, as well as the capacity and resources to conduct all trial processes and data completeness. These measures aimed to assess how effectively participants were recruited and retained throughout the study. | - The primary outcome measure was the rate of eligibility per month, while secondary outcome measures included rates of recruitment, enrolment per month, retention, adherence, and data completeness. These measures were assessed to evaluate the feasibility of the study design and the effectiveness of the recruitment strategies. - Validated scales were utilized to assess clinical outcome measures, including the Montgomery-Asberg Depression Rating Scale (MADRS), the Depression, Anxiety and Stress Scale, and the WHO Quality of Life BREF Instrument. This approach ensured that the data collected was reliable and could be effectively analyzed to determine the impact of probiotic supplementation on various health parameters. | - In the placebo (PLC) group, participants reported acute upper airway infections (including COVID-19; n=3) and one case of urinary tract infection (n=1). - In the probiotic (PRO) group, adverse events included diarrhea (n=2), one case of upper airway infection (n=1), and one exacerbation of allergic asthma (n=1). No serious adverse events were observed in either group. | - The follow-up duration for participants in the study was 60 days, during which they were monitored for adherence to the intervention and completion of assessments related to depressive, anxiety, and stress symptoms, as well as various metabolic and inflammatory parameters. - Participants were required to complete both blood and stool sample collections during this 60-day period, and the retention ratio for the study was reported to be 80%, indicating a high level of participant engagement throughout the follow-up duration. | - The effect size for the comparison of the MADRS score between the probiotic group (PRO-D) and the placebo group (PLC-D) was calculated to be 0.51, indicating a moderate effect of the probiotic intervention on depressive symptoms. This suggests that the probiotic group experienced a more significant reduction in depressive symptoms compared to the placebo group. - The power of the study was reported to be 0.49, which is below the desired threshold of 0.8. To achieve this level of power, it was estimated that approximately 240 subjects would be needed, with 60 participants in each subgroup, highlighting the need for a larger sample size in future studies to better assess the treatment effect. | - The p-value reported for the retention ratio between the probiotic group and the placebo group was 0.79, indicating no statistically significant difference in retention between the two groups during the study. - A significance level was set at p < 0.05 for the statistical procedures used in the study, which included tests such as the U-Mann Whitney test, Kruskal-Wallis test, and repeated measures ANOVA to assess inter-group differences and changes over time. |  |
| 33 | Masakazu, Tamai., at al,2023 | The study utilizes a mouse model to investigate the relationship between constipation and acne vulgaris, focusing on the gut-skin axis. |  |  | - The study utilized a mouse model to investigate the effects of constipation on the gut-skin axis in relation to acne vulgaris. |  | - The study focuses on acne vulgaris, a common skin condition characterized by the presence of pimples, blackheads, and cysts, primarily affecting adolescents and young adults. |  |  | - The primary outcome of the study was to investigate the impact of constipation on the gut-skin axis and its relationship with acne vulgaris in a mouse model. | - The study investigates the relationship between constipation and the gut-skin axis, specifically how constipation may exacerbate skin conditions like acne vulgaris in a mouse model. |  |  |  |  |  |  |
| 34 | Else Schneider, at al,2023 | The study population consisted of 60 patients diagnosed with major depressive disorder (MDD), who were undergoing treatment as usual for their depression. Participants were required to have Hamilton Rating Scale for Depression (HAM-D) scores greater than 7, indicating mild to moderate depression. | - The study is a secondary analysis of a randomized controlled trial (RCT) conducted in Basel, Switzerland, and data were collected between March 2017 and January 2020. | - The duration of the probiotic supplementation in the study was 31 days, which is equivalent to 4 weeks. | - The study initially included 60 patients with major depressive disorder (MDD), but only 43 patients were included in the modified intention-to-treat analysis after excluding noncompliant participants and those with missing assessments at the post-intervention time point. | - The probiotic species used in the study included **Lactobacillus salivarius subsp. Thermophilus**, which was administered at a high dose resulting in a daily intake of **900 billion CFU** (colony-forming units). | - The patients in the study were diagnosed with Major Depressive Disorder (MDD), | - The control group consisted of participants who received a placebo containing maltose but no bacteria, visually indistinguishable from the probiotic supplement. |  | - The primary outcome of the study was the improvement in cognitive symptoms, specifically measured through participant scores on the Verbal Learning Memory Test (VLMT), which showed significantly improved immediate recall in the probiotic group immediately after the intervention. | - The secondary outcome of the study included the assessment of cognitive functions, specifically focusing on verbal episodic memory, which was measured using the Verbal Learning Memory Test (VLMT). The results indicated a significantly improved immediate recall in the probiotic group immediately after the intervention, suggesting that probiotic supplementation may enhance cognitive performance in patients with major depressive disorder (MDD). | - The study employed a modified intention-to-treat (ITT) analysis to assess the treatment effect of probiotics on cognition and related biological markers, which allows for the inclusion of all randomized participants while accounting for noncompliance and dropouts. - Mixed model analyses were utilized to evaluate changes in brain-derived neurotrophic factor (BDNF) levels and cognitive test scores over time, including group x time interactions, to determine the significance of differences between the probiotic and placebo groups across multiple assessment points. | - Patients had the right to withdraw from the study without giving a reason, and adverse events that challenged their health, severe protocol violations, or administrative troubles resulted in withdrawal from the study. - The study did not provide specific details on the nature or frequency of adverse events reported by participants during the trial. | - The follow-up assessment for participants occurred 4 weeks after the intervention, during which all measures were completed except for brain imaging. - This follow-up aimed to evaluate the long-lasting effects of the probiotic supplementation on cognitive symptoms and brain function in patients with major depressive disorder. | - The effect size (Cohen's d) was calculated to analyze changes in cognition and BDNF levels between the probiotic and placebo groups. The results indicated a mean change score of BDNF levels from baseline to post-intervention in the probiotics group of -0.28, suggesting a slight increase, while the placebo group showed a decrease of 1.53. However, the group difference in BDNF levels was not significant, with Cohen's d reported as -0.14. - In the context of cognitive performance, the study utilized 2-sample t tests to compare change scores from baseline to post-intervention between groups, which included calculating the corresponding effect size (Cohen's d) to assess the magnitude of the differences observed in cognitive tasks, particularly focusing on the immediate recall in the Verbal Learning Memory Test (VLMT) where significant improvements were noted in the probiotic group. | - The p-value for the comparison of change scores in BDNF levels between the probiotics group and the placebo group was 0.45, indicating no significant difference between the two groups. - For the analysis of the time × group interaction regarding BDNF levels over all three time points, the p-value was 0.12, which also suggests that there were no significant differences between the groups over time. | - The study discusses the heterogeneity of results regarding the effects of probiotics on cognition, suggesting that this variability may stem from differences in patient samples and methodological approaches across various studies. This includes the variety of cognitive tasks used to assess cognition, which may lead to domain-specific probiotic effects. - A meta-analysis that included 1500 individuals from 22 different studies found inconsistent results regarding probiotic effects on cognition, indicating that methodological differences, such as the type of probiotic used (multistrain vs. monostrain) and the duration of the intervention, could contribute to the observed heterogeneity in findings. |
| 35 | Braden, T.,at al,2022 | The study involved a pediatric population, specifically children aged 3 to 17 years, who were experiencing constipation. | - The study was a randomized, placebo-controlled trial conducted to evaluate the efficacy of a synbiotic on increasing weekly bowel movements in constipated children, with a sample size of 64 children aged 3-17 | - The duration of the study was 84 days, during which the children received either the synbiotic treatment or a placebo. | - The study involved a total of sixty-four children aged between 3 to 17 years. | - The synbiotic treatment in the study included nine microbial strains, which contributed to the increased abundance of bifidobacteria in the gut microbiome of children receiving the intervention compared to those on placebo.  - All administered probiotic species were found to be enriched in the gut microbiome of the intervention group, indicating a successful incorporation of these microbial strains into the children's gastrointestinal | - The study focused on constipated children aged 3-17 years, evaluating the efficacy of a synbiotic intervention to increase weekly bowel movements (WBMs) in this pediatric population. | - The control group consisted of 31 children who received a placebo during the 84-day study period. |  | - The primary outcome of the study was to evaluate the change from baseline in weekly bowel movements (WBMs) in constipated children who received the synbiotic treatment compared to those who received a placebo. | - The primary outcome of the study was the change from baseline in weekly bowel movements (WBMs) in the treatment group compared to the placebo group. The treatment with the synbiotic significantly increased the number of WBMs in children with low baseline WBMs, indicating an improvement in constipation. | - The study utilized a randomized, placebo-controlled design to evaluate the efficacy of a synbiotic on increasing weekly bowel movements (WBMs) in constipated children, allowing for a comparison between the treatment and placebo groups. - Statistical significance was determined using p-values, with a p-value of less than 0.05 indicating a significant increase in the number of WBMs in the treatment group compared to the placebo group, and a p-value of 0.00074 indicating a significant relationship between low baseline microbial richness and improvements in constipation. | - The abstract does not mention any adverse events reported during the study, focusing instead on the efficacy of the synbiotic treatment in increasing weekly bowel movements in constipated children. - Since the study was a randomized, placebo-controlled trial, it is implied that safety and adverse events would typically be monitored, but specific details regarding any adverse events are not provided in the text. | - The follow-up duration for the study was 84 days, during which the children received either the synbiotic treatment or a placebo. - Stool microbiota samples were collected at both baseline and the completion of the 84-day intervention to analyze the effects of the treatment on bowel movements and gut microbiome composition. | - The study evaluated the efficacy of a synbiotic on increasing weekly bowel movements (WBMs) in constipated children, with treatment resulting in a statistically significant increase in WBMs (p < 0.05) compared to the placebo group. However, the specific effect size (Cohen’s d) was not provided in the abstract. - The findings indicated that low baseline microbial richness in the treatment group was a significant predictor of improvements in constipation (p = 0.00074), suggesting that the synbiotic intervention had a meaningful impact on digestive health, although the exact effect size was not quantified. | - The study found that treatment with the synbiotic significantly increased the number of weekly bowel movements (WBMs) in children with low baseline WBMs, with a p-value of less than 0.05, indicating statistical significance. - Additionally, the improvement in constipation was associated with a p-value of 0.00074, suggesting a strong correlation between low baseline microbial richness in the treatment group and the positive response to the synbiotic intervention. |  |
