# Supplementary material for: Long-term safety and clinical effects of probiotic supplementation in at-risk populations: a systematic review and meta-analysis
Source: Front Nutr. 2026 Aug 18;13:1895136. doi: 10.3389/fnut.2026.1895136 (PMC13529487; doi:10.3389/fnut.2026.1895136)
Supplement: Supplementary file 3 [file Table_3.docx]

## Supplementary Table S3. Performance metrics of simulation-based estimators (Bootstrap resampling, Monte Carlo simulation, and Bayesian MCMC) in assessing pooled probiotic effects.

| ****Estimator / Method**** | ****Mean RR**** | ****Bias**** | ****Variance**** | ****MSE**** | ****Coverage (95%)**** | ****Notes**** |
| --- | --- | --- | --- | --- | --- | --- |
| Bootstrap (10,000 resamples) | 1.24 | 0.00 | 0.012 | 0.012 | 94.8% | Stable, consistent with main meta-analysis |
| Monte Carlo Simulation | 1.25 | +0.01 | 0.011 | 0.012 | 95.2% | No systematic bias; robust under study-level variance |
| Bayesian MCMC (posterior) | 1.23 | -0.01 | 0.013 | 0.014 | 95.5% | Results overlap with frequentist estimates |

**Abbreviations:** RR, risk ratio; MSE, mean squared error; MCMC, Markov chain Monte Carlo.
