# Supplementary material for: Long-term safety and clinical effects of probiotic supplementation in at-risk populations: a systematic review and meta-analysis
Source: Front Nutr. 2026 Aug 18;13:1895136. doi: 10.3389/fnut.2026.1895136 (PMC13529487; doi:10.3389/fnut.2026.1895136)
Supplement: Supplementary file 4 [file Table_4.docx]

**Supplementary Table S4 — Outcome Definitions for “Clinical Improvement”**

| Study | Outcome Domain | Instrument | Definition of Improvement | Pre‑specified in Original Trial? |
| --- | --- | --- | --- | --- |
| Study A | Gastrointestinal symptoms | **UCLA Gastrointestinal Symptom Score (UCLA‑GIT)** | ≥ **0.5‑point reduction** in the total UCLA‑GIT score, consistent with the minimal clinically important difference (MCID) reported in GI symptom trials | Yes |
| Study B | IBS symptom severity | **IBS Severity Scoring System (IBS‑SSS)** | ≥ **50‑point reduction** from baseline, the established MCID for IBS‑SSS | Yes |
| Study C | Anxiety / Depression | **HADS‑A / HADS‑D** (Hospital Anxiety and Depression Scale) | ≥ **1.5‑point reduction** in either subscale, consistent with validated MCID thresholds | Yes |
| Study D | Microbiota composition | **Alpha‑diversity (Shannon index)** and **relative abundance of beneficial taxa** | Improvement defined as **increase in alpha‑diversity** and/or **≥10% relative increase** in predefined beneficial genera (e.g., Bifidobacterium, Lactobacillus) | Post‑hoc |
| Study E | Composite GI + microbiota | Mixed GI symptom scale + microbiota indices | Improvement defined as **symptom reduction ≥ MCID** and **favorable shift in microbiota composition** | Partially pre‑specified |
